# RTKN-1/Rhotekin shields endosome-associated F-actin from disassembly to ensure endocytic recycling

**DOI:** 10.1083/jcb.202007149

**Published:** 2021-04-12

**Authors:** Yanling Yan, Shuai Liu, Can Hu, Chaoyi Xie, Linyue Zhao, Shimin Wang, Wenjuan Zhang, Zihang Cheng, Jinghu Gao, Xin Fu, Zhenrong Yang, Xianghong Wang, Jing Zhang, Long Lin, Anbing Shi

**Affiliations:** 1 Department of Biochemistry and Molecular Biology, School of Basic Medicine, Tongji Medical College, Huazhong University of Science and Technology, Wuhan, Hubei, China; 2 Cell Architecture Research Institute, Huazhong University of Science and Technology, Wuhan, Hubei, China

## Abstract

Cargo sorting and the subsequent membrane carrier formation require a properly organized endosomal actin network. To better understand the actin dynamics during endocytic recycling, we performed a genetic screen in *C. elegans* and identified RTKN-1/Rhotekin as a requisite to sustain endosome-associated actin integrity. Loss of RTKN-1 led to a prominent decrease in actin structures and basolateral recycling defects. Furthermore, we showed that the presence of RTKN-1 thwarts the actin disassembly competence of UNC-60A/cofilin. Consistently, in RTKN-1–deficient cells, UNC-60A knockdown replenished actin structures and alleviated the recycling defects. Notably, an intramolecular interaction within RTKN-1 could mediate the formation of oligomers. Overexpression of an RTKN-1 mutant form that lacks self-binding capacity failed to restore actin structures and recycling flow in *rtkn-1* mutants. Finally, we demonstrated that SDPN-1/Syndapin acts to direct the recycling endosomal dwelling of RTKN-1 and promotes actin integrity there. Taken together, these findings consolidated the role of SDPN-1 in organizing the endosomal actin network architecture and introduced RTKN-1 as a novel regulatory protein involved in this process.

## Teaser

Yan et al. identify *C. elegans* RTKN-1 as a novel endocytic recycling regulator that impedes UNC-60A/cofilin-mediated endosomal actin disassembly. Further evidence indicates that the self-binding capacity of RTKN-1 is indispensable for this functionality and SDPN-1/Syndapin acts to direct the proper residency of RTKN-1 in recycling endosomes.

## Introduction

Endocytosed membrane proteins are usually delivered to early endosomes, where initial cargo sorting occurs. After that, some membrane proteins will be sent back to the cell surface via the recycling pathway (Grant and Donaldson, 2009; Hsu and Prekeris, 2010; Cullen and Steinberg, 2018; Liu et al., 2018). Increasing evidence has suggested that actin structures play crucial roles in concerted regulation of endocytic recycling (Wang et al., 2016; Delevoye et al., 2016; Puthenveedu et al., 2010; Gong et al., 2018; Gleason et al., 2016). For instance, when EGFR and MT1-MMP are mutated and thus lose actin-binding capacity, their recycling transport is severely impaired (MacDonald et al., 2018). Conversely, the insertion of an actin-binding module into a cargo destined to lysosomes leads to the missorting of the cargo into the recycling route (MacDonald et al., 2018). In agreement with these observations, WASH and Arp2/3 can be localized onto endosomes to administer local actin dynamics, therefore promoting the biogenesis of tubular membrane carrier (Puthenveedu et al., 2010; Simonetti and Cullen, 2019; Gong et al., 2018). WASH-mediated actin assembly was also suggested to assist dynamin-mediated membrane remodeling (Derivery et al., 2009). Our recent mechanistic inquiry showed that CAMSAPs/Patronin homologue PTRN-1 resides in the sorting endosomes and unlocks the self-inhibitory configuration of CYK-1, hence facilitating actin polymerization during recycling (Gong et al., 2018). Actin disassembly factor ADF/cofilin has also been implicated in endocytic trafficking. During metastasis, a decrease of cofilin phosphorylation disturbed the endocytosis of EGFR, and this defect could be alleviated by the overexpression of a cofilin phosphomimicking (S3E) mutant (de Graauw et al., 2014). In *Caenorhabditis elegans*, UNC-60A/cofilin promotes actin dynamics in the invadopodia of anchor cells. In the absence of UNC-60A, invadopodia membrane recycling is undermined (Hagedorn et al., 2014). Various actin-binding proteins, including myosin and tropomyosin, have been shown to compete with cofilin for F-actin binding and thus affect the severing efficacy of cofilin (Ngo et al., 2016; Ono and Ono, 2002; Christensen et al., 2017). Specifically, the motor domain of myosin inhibits the generation of cofilin clusters along F-actin (Ngo et al., 2016). Likewise, tropomyosin forms head-to-tail multimers along F-actin and displaces cofilin (Li et al., 2011; Meiring et al., 2018; Jansen and Goode, 2019).

Rhotekin/RTKN was initially identified as a binding protein of RhoA and RhoC in mice (Reid et al., 1996). Rhotekin contains an N-terminal Rho-binding domain (RBD) and a central pleckstrin homology (PH) domain, followed by two proline-rich motifs (PRMs) and a PDZ domain recognizing X(S/T)XV-COOH consensus motif (Ito et al., 2018). The PH domain possesses an affinity for phosphoinositide and aids in localizing proteins to specific membranous structures (Garcia et al., 1995; Mao et al., 2009). Recently, Rhotekin was implicated in the interaction with the Src homology 3 (SH3) domain of vinexin/SORBS3 (Ito et al., 2018). The vinexin SH3 domain interacts with the PRM and recruits Rhotekin to the midbody during cell division (Nagata et al., 2009; Chang and Huang, 2017). Also, through interaction between the C-terminus and the PDZ domain of LIN-7B, Rhotekin can be located at the junctions of MDCK cells and participate in establishing or maintaining cell polarity (Sudo et al., 2006). It is worth noting that RTKN2/Rhotekin was upregulated in colon cancer cells. In the absence of RTKN2, the proliferation rate slowed down, and the cell cycle progression was affected (Pang et al., 2017). Furthermore, the Rho-Rhotekin signaling pathway has been involved in septin cytoskeleton assembly, which is closely correlated to cancer development (Ito et al., 2005).

Here, we found that lack of RTKN-1/Rhotekin undermined basolateral recycling transport and actin integrity in the *C. elegans* intestine. Specifically, in the absence of RTKN-1, newly formed membrane carriers still emanated from recycling endosomes but failed to move along actin remnants and finally retracted. Subsequent assays revealed that RTKN-1 was capable of impeding UNC-60A–mediated actin disassembly. Accordingly, RTKN-1 deficiency–induced recycling defect was alleviated by UNC-60A knockdown. Further testing showed that the interaction between RBD and the C-terminal segment (CT) is required for the oligomerization of RTKN-1. The overexpressed RTKN-1ΔCT could neither defer actin disassembly nor alleviate the recycling defects in *rtkn-1* mutants. Finally, we identified that recycling regulator SDPN-1/Syndapin interacted with PRM and thus directed the proper dwelling of RTKN-1 in recycling endosomes. Together, these findings led to a model of actin homeostasis during endocytic recycling where RTKN-1 stabilizes endosome-associated F-actin, promoting the formation and directional movement of recycling membrane carriers.

## Results

### RTKN-1 is required to sustain actin integrity in the intestinal epithelia

Dynamic actin architecture facilitates membrane carrier biogenesis and movement during endocytic recycling (Campellone and Welch, 2010; Wang et al., 2016; Gong et al., 2018). To better understand the mechanisms underlying recycling endosome–associated actin dynamics, we performed a two-step RNAi genetic screen in *C. elegans*, attempting to identify new regulators in this process (see Materials and methods for detail). We used recycling cargos to follow the recycling aberration in the intestinal epithelia (Chen et al., 2006). Next, we sought to recognize actin regulators among the subset of genes whose expression knockdown leads to recycling defects. To this end, we identified C07B5.4 as a candidate, which encodes a homologue of Rhotekin. Notably, although Rhotekin is implicated in the septin assembly (Ito et al., 2005), the interaction between Rhotekin and actin dynamics remains unknown.

**Figure 1.**
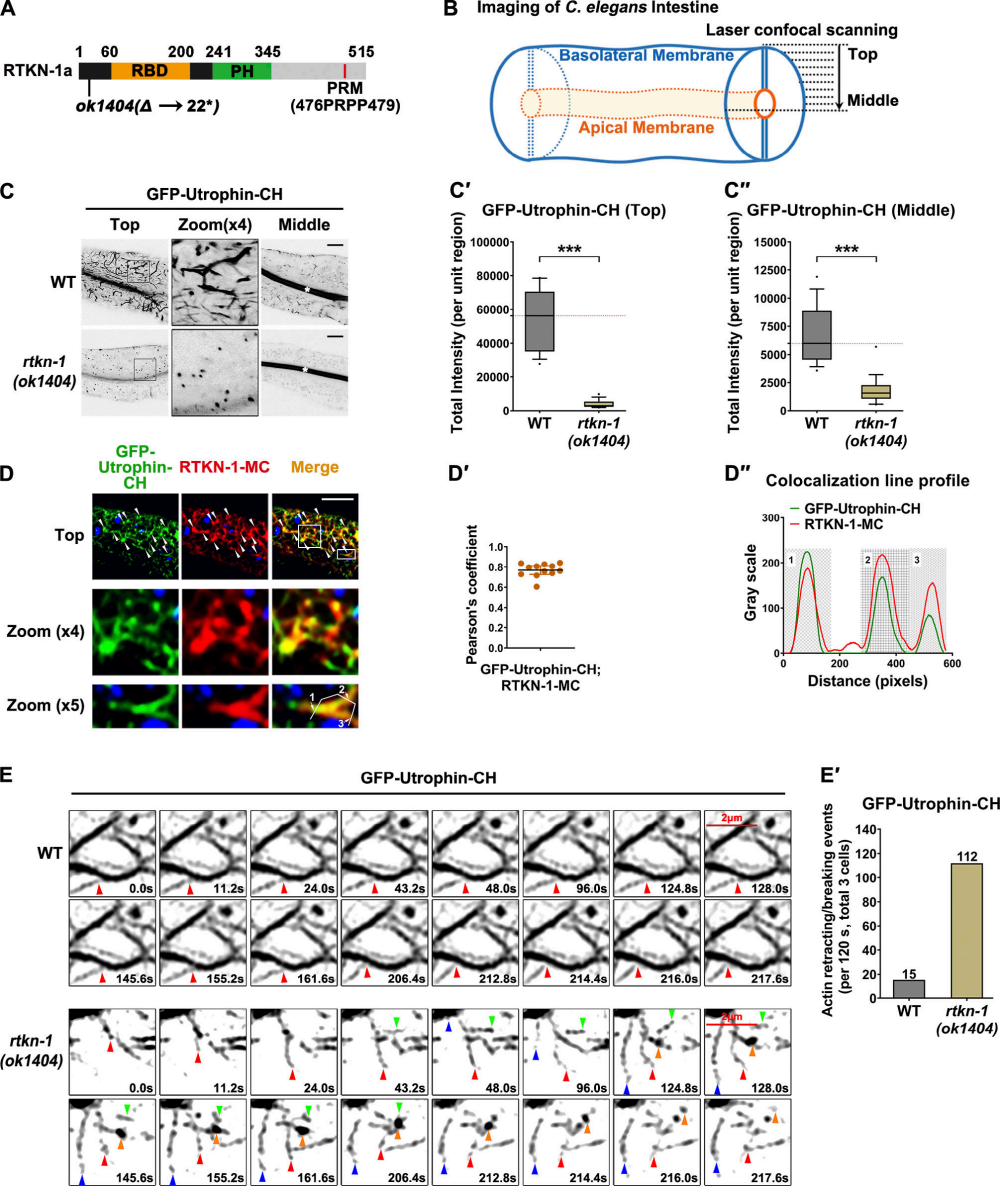
**RTKN-1 is required for actin integrity in the intestinal epithelia. (A)** Domain architecture of RTKN-1a and allele information of *rtkn-1(ok1404)*. Δ indicates deletion, and * indicates stop codon. **(B)** A diagram of *C. elegans* intestine and laser confocal scanning. **(C–C′′)** Confocal images showing GFP-Utrophin-CH–labeled actin structures in the intestinal cells. White asterisks in the panels indicate intestinal lumen. Box-and-whisker plots (*n* = 18 cells): 10–90th percentile; dots, outliers; red midline, median of WT; boundaries, quartiles. ***, P < 0.001 by Mann-Whitney test. **(D and D′)** Confocal images showing the colocalization between GFP-Utrophin-CH and RTKN-1-mCherry. Arrowheads indicate structures labeled by both GFP and mCherry. DAPI channel (blue color) indicates broad-spectrum intestinal autofluorescence. Pearson's correlation coefficient was calculated; error bar is 95% CI (*n* = 12 animals). **(D′′)** Line scan profile of GFP and mCherry signals. **(E and E′)** Live cell fluorescence images showing the dynamics of GFP-Utrophin-CH–labeled actin structures. A total of three intestinal cells of each genotype were sampled. Scale bars, 10 µm. MC, mCherry.

**Figure S1.**
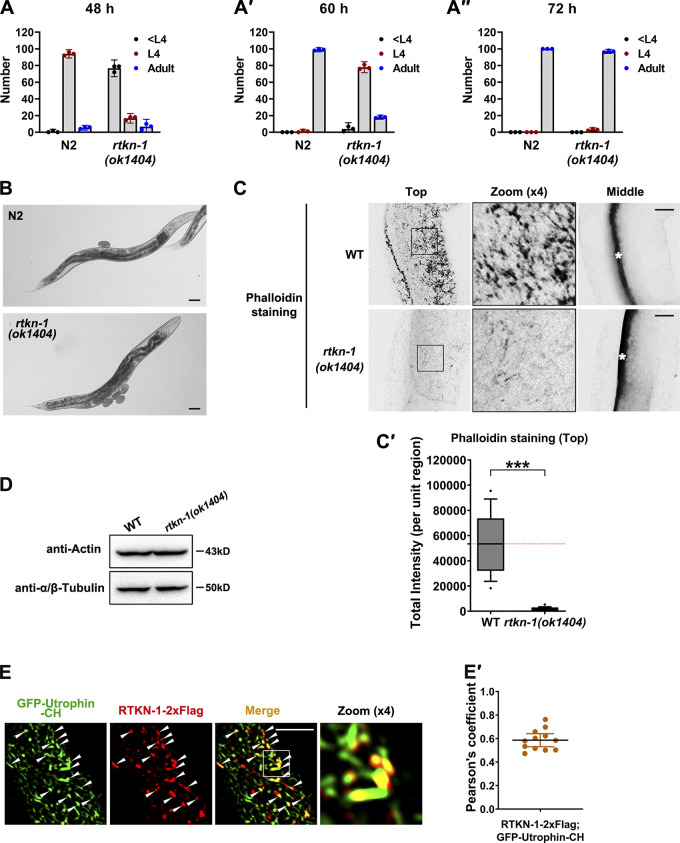
**RTKN-1 could play a role during development, and loss of RTKN-1 impairs actin integrity. (A–A′′)** Measurement of growth time from the L1 larval stage to the young adult stage. **(B)** Differential interference contrast microscopy showing WT and mutant animals. Scale bars, 50 µm. **(C and C′)** Confocal images showing Alexa Fluor 488–phalloidin staining in the fixed intestinal cells. White asterisks in the middle focal panels indicate intestinal lumen. Box-and-whisker plots (*n* = 18 cells): 10–90th percentile; dots, outliers; red midline, median of WT; boundaries, quartiles. ***, P < 0.001 by Mann-Whitney test. **(D)** Loss of RTKN-1 did not interfere with expression of intestinal actin. **(E and E′)** Confocal images showing the colocalization between GFP-Utrophin-CH and endogenous RTKN-1. The white arrowheads indicate structures labeled by both GFP and mCherry. Pearson's correlation coefficients were calculated; error bars are 95% CIs (*n* = 12 animals). Scale bars, 10 µm.

Here, we named C07B5.4 as RTKN-1 with reference to the nomenclature of its mammalian homologue and employed an *rtkn-1(ok1404)* null allele bearing a 1,345-nucleotide intragenic deletion (outcrossed three times), which led to an early nonsense mutation (Fig. 1 A). By measuring growth time from the L1 larval stage to the young adult stage, we observed that 60 h after the L1 stage, ∼80% of *rtkn-1* mutants were still at the L4 stage (Fig. S1, A–A"), highlighting that RTKN-1 could play a role during development. Indeed, the intestine of rtkn-1 mutant adults appeared to be thinner (Fig. S1 B). To present the location of fluorescent proteins, the imaging plane close to the basal membrane of the intestinal cells was defined as “top,” and the imaging plane where the apical membrane and lumen can be observed was defined as “middle” (deep cytosol; Fig. 1 B). The utrophin calponin homology (CH) domain (Utrophin-CH) bears a binding specificity for F-actin and has been adopted as a faithful actin filaments probe in *C. elegans* (Mizumoto and Shen, 2013; Chia et al., 2012). We validated that Utrophin-CH–labeled actin structures near the basolateral membrane were reduced in *rtkn-1* mutants, leaving a few cytoplasmic punctate structures (Fig. 1, C–C"). Phalloidin has been used to label endogenous actin in *C. elegans* (Gong et al., 2018). Likewise, while the level of actin in *rtkn-1* mutant intestine is comparable to that of WT animals (Fig. S1 D), phalloidin-stained actin structures were severely reduced upon loss of RTKN-1 (Fig. S1, C and C'), corroborating that RTKN-1 plays a critical role in maintaining the integrity of actin structures in the intestinal epithelia.

RTKN-1 is broadly expressed in *C. elegans* (Birchall et al., 1995; Spencer et al., 2011), including intestine and coelomocyte. We prepared a CRISPR-based 2xFlag knockin strain to examine RTKN-1 level via Western blot (Table S1). The results showed that the endogenous RTKN-1 could be readily detected (Fig. S4 E). Moreover, we observed that endogenous RTKN-1 resides on the actin structures in the intestine (Fig. S1, E and E'). To obtain an ideal fluorescent signal-to-noise ratio, we deployed the integrated low-expression transgenic strains (intestinal *vha-6* promoter driven) in the subsequent studies. Consistent with the localization of the endogenous protein, RTKN-1-mCherry maintained a notable dwelling on the actin structures near the basolateral membrane (Fig. 1, D–D").

To probe the details underlying the actin diminishment phenotype, we tracked the dynamic changes of Utrophin-CH–labeled actin structures. In this respect, we noted that intestinal actin structures are relatively stable, with occasional attachment, detachment, and wobble (Fig. 1, E and E'). However, in RTKN-1–depleted cells, actin structures are very dynamic and frequently elongate, retract, break, and fade out (Fig. 1, E and E'), consolidating the role of RTKN-1 in maintaining actin integrity.

### RTKN-1 is localized in basolateral recycling endosomes

**Figure 2.**
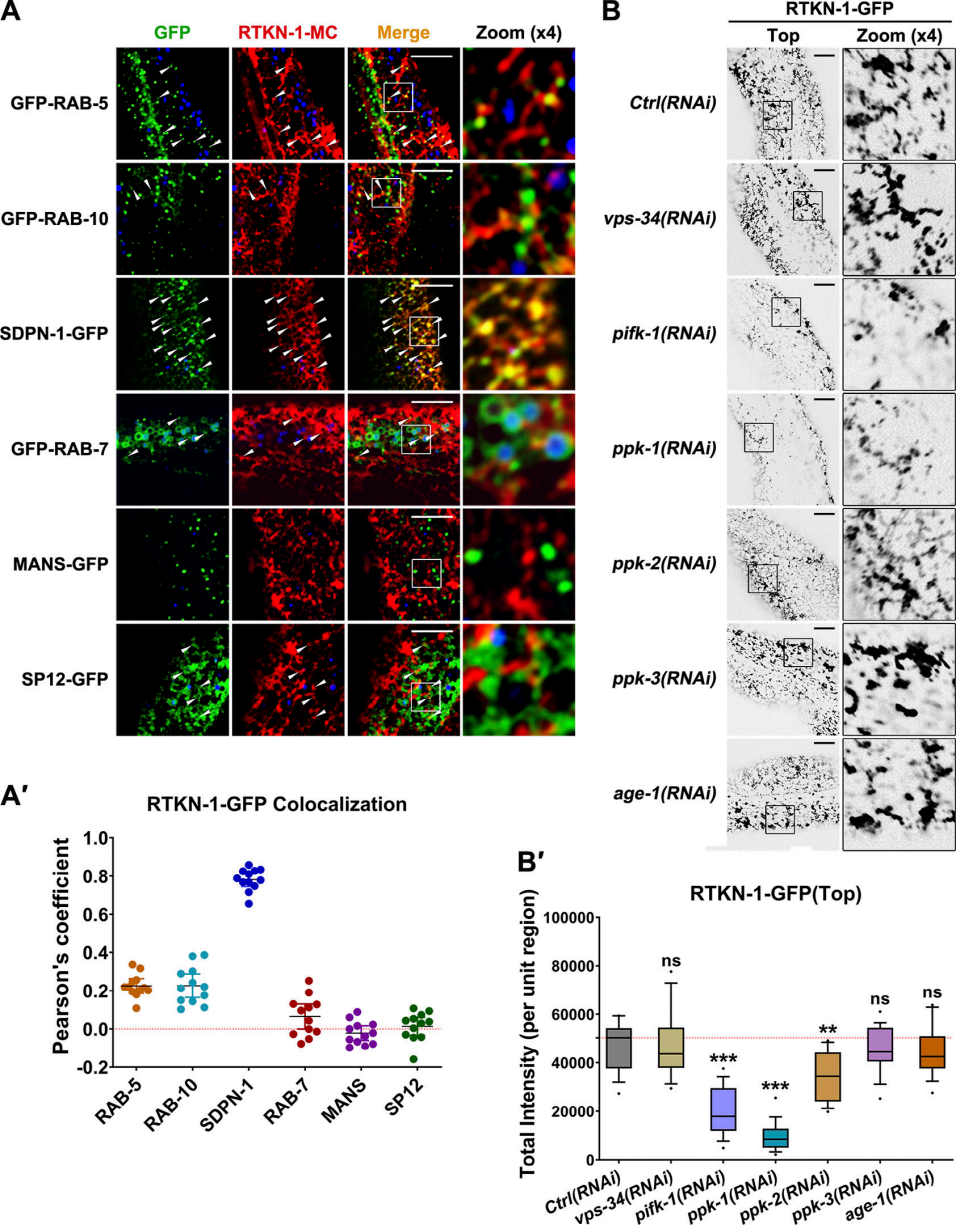
**RTKN-1 resides in basolateral recycling endosome. (A and A′)** Confocal images showing colocalization between RTKN-1-mCherry and organelle markers. Arrowheads indicate structures labeled by both GFP and mCherry. DAPI channel (blue color) indicates broad-spectrum intestinal autofluorescence. Pearson's correlation coefficients were calculated; error bars are 95% CIs (*n* = 12 animals). **(B and B′)** Confocal images showing RTKN-1-GFP distribution upon knock down of phosphatidylinositol kinases. Box-and-whisker plots (*n* = 18 cells): 10–90th percentile; dots, outliers; red midline, median of WT; boundaries, quartiles **, P < 0.01; ***, P < 0.001, by one-way ANOVA followed by Dunn’s post hoc multiple comparison test. Scale bars, 10 µm. Ctrl, control; MC, mCherry.

**Figure S2.**
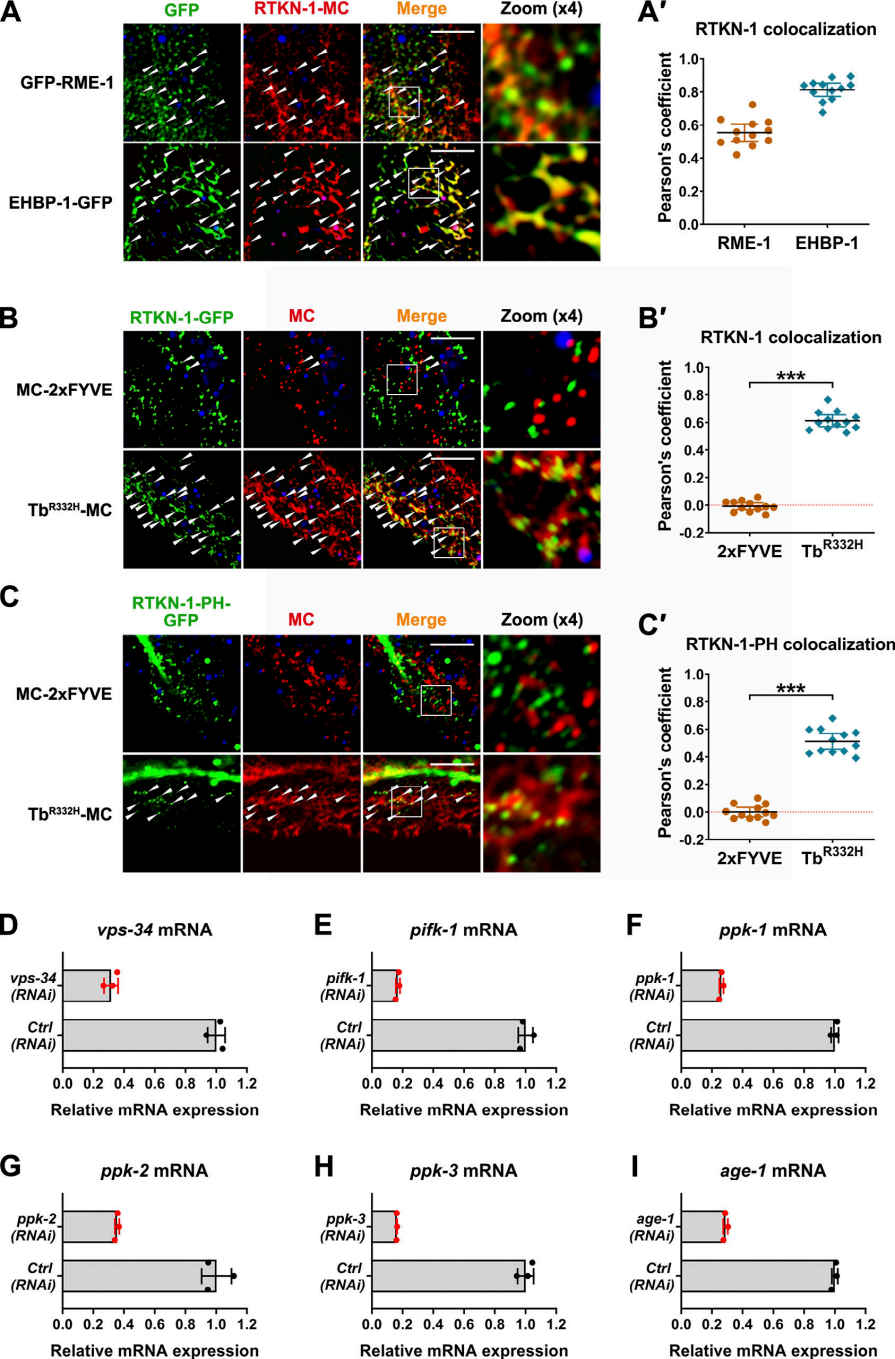
**RTKN-1 is localized in recycling endosomes. (A and A′)** Confocal images showing colocalization between RTKN-1-mCherry and recycling endosome markers. **(B–C′)** Confocal images showing colocalization between GFP-tagged RTKN-1 or RTKN-1-PH and endosome markers. In these panels, the DAPI channel (blue color) indicates broad-spectrum intestinal autofluorescence. Pearson's correlation coefficients were calculated; error bars are 95% CIs (*n* = 12 animals). ***, P < 0.001 by Mann-Whitney test. **(D–I)** mRNA level measurement showing RNAi efficiency of phosphatidylinositol kinases. The white arrowheads indicate structures labeled by both GFP and mCherry. Scale bars, 10 µm. Ctrl, control; MC, mCherry; Tb, Tubby.

Our screen indicated that RTKN-1 is indispensable for basolateral recycling. To assess RTKN-1 subcellular localization, we performed colocalization assays with a set of organelle markers (Chen et al., 2006; Shi et al., 2012). RTKN-1-mCherry mainly resided in tubular and punctate structures and exhibited a limited colocalization with early endosome marker GFP-RAB-5 and clathrin-independent cargo recycling regulator RAB-10 (Fig. 2, A and A'; Shi et al., 2010). Likewise, there was no significant overlap of RTKN-1-mCherry with late endosome marker GFP-RAB-7, Golgi marker α-mannosidase II-GFP (MANS-GFP), and ER marker signal peptidase (SP12-GFP; Fig. 2, A and A'). However, recycling endosome markers (SDPN-1-GFP, GFP-RME-1, and EHBP-1-GFP) displayed a notable colocalization with RTKN-1-mCherry (Fig. 2, A and A'; and Fig. S2, A and A'). Of note, mammalian recycling endosome consists of membrane tubules and discrete vesicles (Maxfield and McGraw, 2004). Similarly, in the *C. elegans* intestine, the recycling endosome network is highly tubular and punctate (Grant et al., 2001). We speculated that these endosomal structures are F-actin associated given the substantial colocalization between RTKN-1 and Utrophin-CH in tubular and punctate structures.

Phosphatidylinositol-3-bisphosphate (PI(3)P) mainly exists in the early endosomes, and PI(4,5)P2 is abundant in the recycling endosomes (Wang et al., 2016; Chen et al., 2018). By using EEA-1 2xFYVE motif and Tubby-PH^(R332H)^ (the reporters of PI(3)P and PI(4,5)P2, respectively; Quinn et al., 2008; Hardie et al., 2015), we found that RTKN-1 maintained a substantial colocalization with Tubby-PH^(R332H)^-mCherry but displayed no overlap with mCherry-2xFYVE (Fig. S2, B and B'). PH domain often exhibits an affinity for PI(4,5)P2 (Martinez Marshall et al., 2019). However, the RTKN-1-PH domain solely resided in punctate structures and partially overlapped with Tubby-PH^(R332H)^-mCherry (Fig. S2, C and C'), suggesting that additional elements within RTKN-1 participate in recycling endosome dwelling. We further validated the implication of PI(4,5)P2 in the localization of RTKN-1 by using RNAi-mediated knockdown of phosphatidylinositol kinases (Fig. S2, D–I; Wang et al., 2016). Loss of PI4-kinase PIFK-1 or PI(5)P-directed PI4-kinase PPK-2 led to a moderate decrease in RTKN-1 labeling, and knockdown of PI(4)P-directed PI5-kinase PPK-1 reduced the endosomal residence of RTKN-1-GFP by ∼80% (Fig. 2, B and B').

### Loss of RTKN-1 leads to basolateral recycling defects

**Figure S3.**
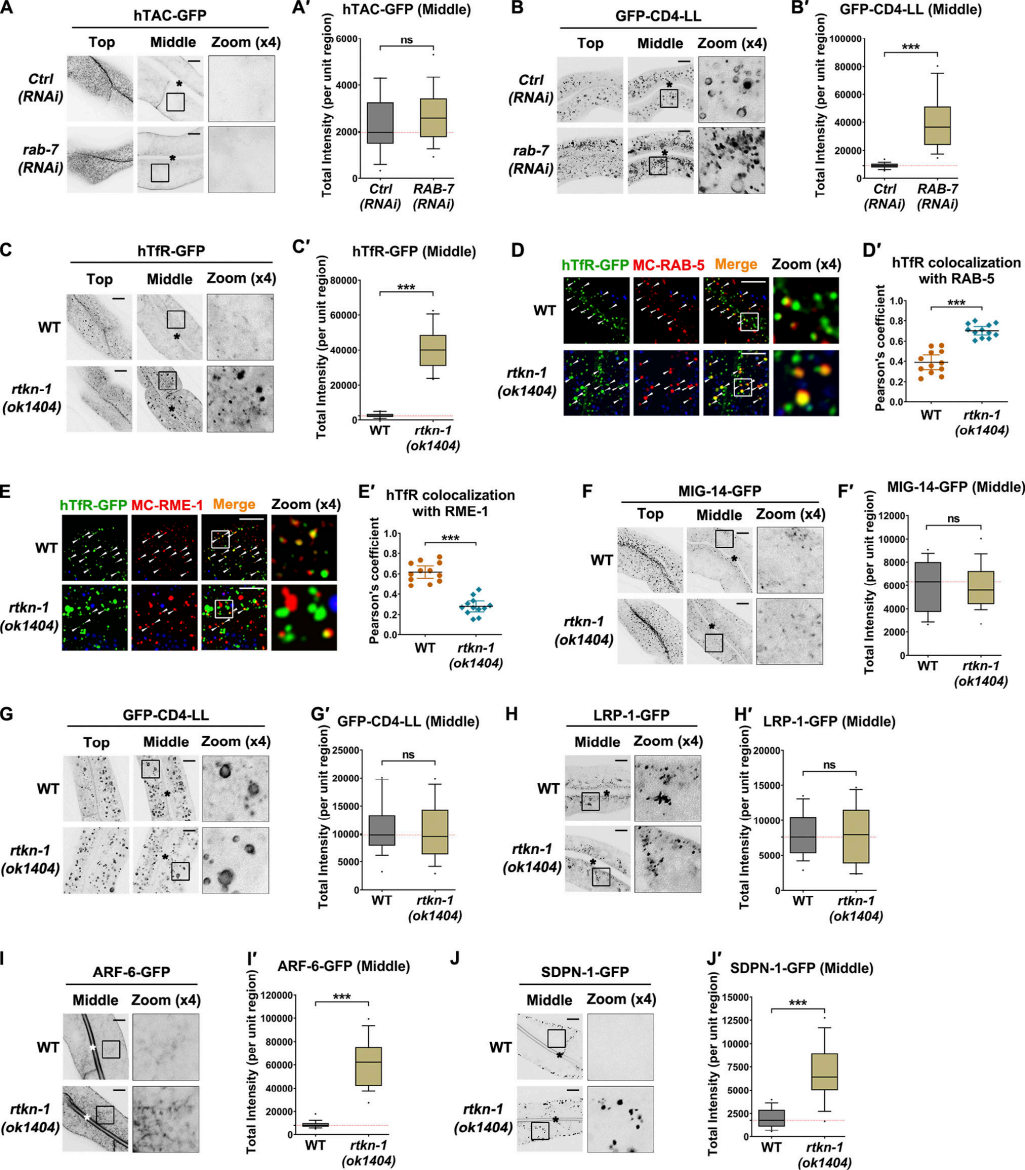
**Loss of RTKN-1 affects basolateral endocytic recycling. (A–C′)** Confocal images showing the localization of CIE recycling cargo hTAC-GFP, clathrin-dependent nonrecycling cargo GFP-CD4-LL, and CDE recycling cargo hTfR-GFP in intestinal cells. Box-and-whisker plots (*n* = 18 cells): 10–90th percentile; dots, outliers; red midline, median of WT; boundaries, quartiles. ***, P < 0.001 by Mann-Whitney test. **(D–E′)** Confocal images showing colocalization between hTfR-GFP and early endosome marker mCherry-RAB-5 or recycling endosome marker mCherry-RME-1. DAPI channel (blue color) indicates broad-spectrum intestinal autofluorescence. Pearson's correlation coefficients were calculated; error bars are 95% CIs (*n* = 12 animals). ***, P < 0.001 by Mann-Whitney test. **(F–H′)** Confocal images showing localization of clathrin-dependent retrograde cargo MIG-14-GFP, clathrin-dependent nonrecycling cargo GFP-CD4-LL, and apical recycling cargo LRP-1-GFP. Box-and-whisker plots (*n* = 18 cells): 10–90th percentile; dots, outliers; red midline, median of WT; boundaries, quartiles. No significant difference by Mann-Whitney test. **(I–J′)** Confocal images showing subcellular localization of recycling endosome markers ARF-6-GFP and SDPN-1-GFP. Box-and-whisker plots (*n* = 18 cells): 10–90th percentile; dots, outliers; red midline, median of WT; boundaries, quartiles. ***, P < 0.001 by Mann-Whitney test. The white arrowheads indicate structures labeled by both GFP and mCherry. The asterisks in the image panels indicate intestinal lumen. Scale bars, 10 µm. Ctrl, control; MC, mCherry.

**Figure 3.**
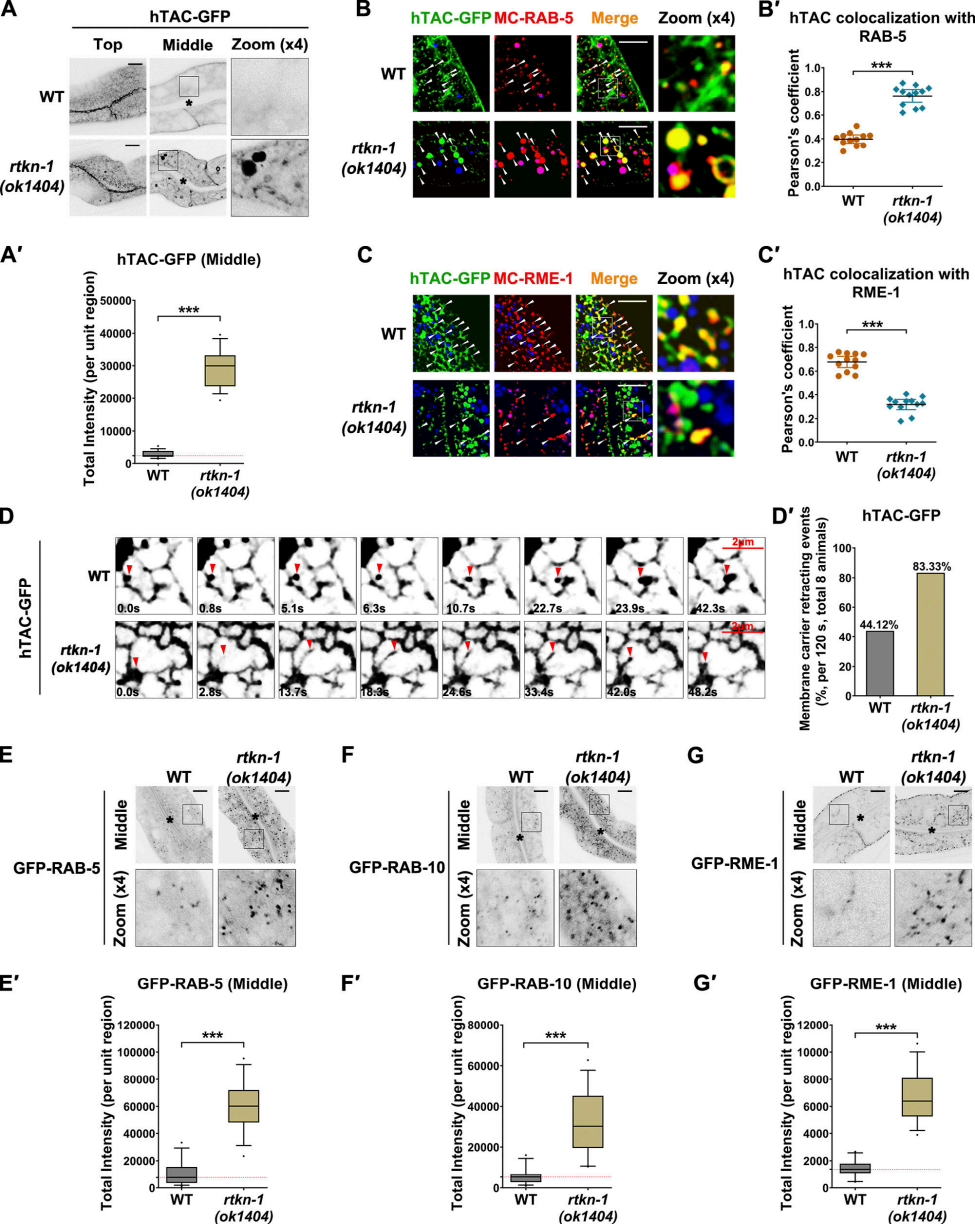
**Loss of RTKN-1 impairs basolateral recycling. (A and A′)** Confocal images showing the subcellular localization of CIE recycling cargo hTAC-GFP in intestinal cells. Box-and-whisker plots (*n* = 18 cells): 10–90 percentile; dots, outliers; red midline, median of WT; boundaries, quartiles. ***, P < 0.001 by Mann-Whitney test. **(B–C′)** Confocal images showing the colocalization between hTAC-GFP and mCherry-tagged RAB-5 or RME-1. Arrowheads indicate structures labeled by both GFP and mCherry. DAPI channel (blue color) indicates broad-spectrum intestinal autofluorescence. Pearson's correlation coefficients were calculated; error bars are 95% CIs (*n* = 12 animals). ***, P < 0.001 by Mann-Whitney test. **(D and D′)** Live cell fluorescence images showing hTAC-GFP–positive endosomal dynamics. A total of eight animals of each genotype were sampled. **(E–G′)** Confocal images showing the localization of early endosome marker GFP-RAB-5, sorting endosome marker GFP-RAB-10, and recycling endosome marker GFP-RME-1 in intestinal cells. Box-and-whisker plots (*n* = 18 cells): 10–90th percentile; dots, outliers; red midline, median of WT; boundaries, quartiles. ***, P < 0.001 by Mann-Whitney test. The black asterisks in the image panels indicate intestinal lumen. Scale bars, 10 µm. MC, mCherry.

To assess the specificity of RTKN-1 in the recycling pathway, we examined the steady-state distribution of various cargo proteins in *rtkn-1* mutants (Chen et al., 2006; Shi et al., 2007). We first validated the intracellular itinerary of hTAC (clathrin-independent recycling cargo, the α-chain of the human interleukin 2 receptor) by assaying the distribution of hTAC-GFP in *rab-7(RNAi)* animals. In the absence of RAB-7, the distribution of hTAC-GFP was unaffected, and clathrin-dependent nonrecycling cargo GFP-CD4-LL overaccumulated in the cytosol (Fig. S3, A–B'; Gu et al., 2013). These results suggested that hTAC-GFP undergoes continuous internalization and recycling with negligible sorting to late endosomes and lysosomes. Likewise, TfR (clathrin-dependent recycling cargo, the human transferrin receptor [hTfR]) has been reported to be continuously internalized and recycled, and its transit to late endosomes is negligible (Sönnichsen et al., 2000). Next, we assessed the recycling implication of RTKN-1 and observed that lack of RTKN-1 caused aggregation of hTAC-GFP and hTfR-GFP in the cytosolic structures (Fig. 3, A and A'; and Fig. S3, C and C'), whereas the distribution of the clathrin-dependent retrograde cargo MIG-14-GFP or nonrecycling cargo GFP-CD4-LL was not affected (Fig. S3, F–G'; Gu et al., 2013; Shi et al., 2009), indicating that RTKN-1 is implicated in the recycling regulation of both clathrin-dependent and clathrin-independent cargos.

To clarify whether RTKN-1 is implicated in apical recycling, we examined the distribution of low-density lipoprotein receptor–related protein 1 (LRP-1)-GFP. LRP-1 localizes to the apical plasma membrane of *C. elegans* hypodermis and can be recycled from endosomes to the plasma membrane (Yochem et al., 2015). In the intestine, LRP-1-GFP mainly resides on the punctate structures around the lumenal apical membrane, and it is rarely observed on the basal side (Fig. S3, H and H'). In the absence of RTKN-1, there was no significant aberration of LRP-1-GFP distribution (Fig. S3, H and H'). Together, these results suggested that RTKN-1 is explicitly required for basolateral recycling.

To assess the functional step of RTKN-1 in the recycling pathway, we compared the localization of recycling cargos with that of the early endosome marker mCherry-RAB-5 and the recycling endosome marker mCherry-RME-1 (Chen et al., 2018; Gao et al., 2020). In the WT animals, hTAC-GFP and hTfR-GFP primarily appeared in mCherry-RME-1–labeled tubular and punctate structures and, to a lesser extent, in mCherry-RAB-5–positive endosomes (Fig. 3, B–C'; and Fig. S3, D–E'). In the absence of RTKN-1, the labeling of hTAC-GFP or hTfR-GFP in recycling endosomes was reduced, while their dwelling in early endosomes was increased (Fig. 3, B–C'; and Fig. S3, D–E'), implicating RTKN-1 in the transit between early endosomes and recycling endosomes. By using live animal imaging, we checked the dynamics of endosomal structures. Normally, hTAC-GFP–labeled membrane tubules are highly dynamic, with frequent extension and detachment (Fig. 3, D and D'). In contrast, in RTKN-1–depleted cells, hTAC-GFP–positive tubules were still able to extend but often retracted (Fig. 3, D and D'), indicative of the impaired biogenesis of recycling membrane carrier (Gao et al., 2020).

Recycling interruption often adversely affects the morphology and/or distribution of endosomes in the recycling pathway (Chen et al., 2006). To further assess the functional position of RTKN-1, we assayed the distribution of early/sorting endosomes in *rtkn-1* mutants. Loss of RTKN-1 resulted in the overaccumulation of GFP-RAB-5– and GFP-RAB-10–positive sorting endosomes (Fig. 3, E–F'), suggesting that RTKN-1 functions at a similar step or downstream of RAB-10. Accordingly, in *rtkn-1(ok1404)* animals, the endosomes labeled by the GFP-tagged recycling regulators RME-1, ARF-6, and SDPN-1 were abnormally deposited in the cytosol (Fig. 3, G and G'; and Fig. S3, I–J'), corroborating that RTKN-1 is imperative for the basolateral recycling.

### RTKN-1 acts independently of the Rho family of GTPases during endocytic recycling

**Figure 4.**
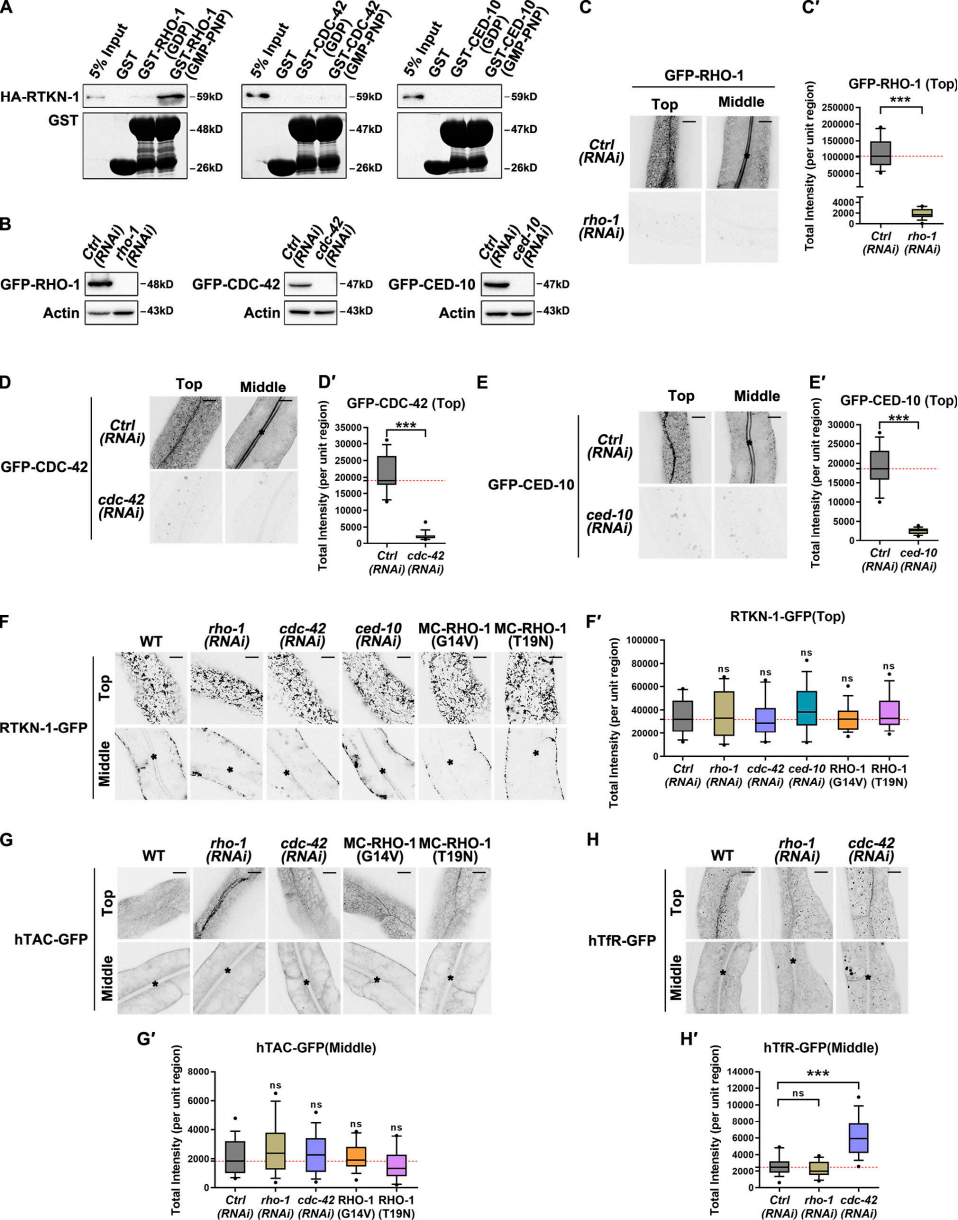
**RTKN-1 acts independently of the Rho family of GTPases during recycling. (A)** Western blot showing GST pulldown with in vitro–translated HA-tagged RTKN-1. RTKN-1 showed an interaction with RHO-1 loaded with GMP-PNP. RTKN-1 displayed no binding to CDC-42 loaded with GDP or GMP-PNP. Also, RTKN-1 displayed no binding to CED-10 loaded with GDP or GMP-PNP. **(B–E′)** Western blot and confocal images showing RNAi-mediated knockdown efficiency of GFP-RHO-1, GFP-CDC-42, and GFP-CED-10. Box-and-whisker plots (*n* = 18 cells): 10–90th percentile; dots, outliers; red midline, median of WT; boundaries, quartiles. ***, P < 0.001 by Mann-Whitney test. **(F–H′)** Confocal images showing subcellular localization of RTKN-1-GFP, hTAC-GFP, and hTfR-GFP in intestinal cells. Box-and-whisker plots (*n* = 18 cells): 10–90th percentile; dots, outliers; red midline, median of WT; boundaries, quartiles. ***, P < 0.001 by one-way ANOVA followed by Dunn’s post hoc multiple comparison test. Scale bars, 10 µm. The black asterisks in the image panels indicate intestinal lumen. Ctrl, control; MC, mCherry.

Studies in mammals identified Rhotekin as an effector of the Rho family of GTPases during cell polarity establishment and neuronal synapse formation (Reid et al., 1996; Ito et al., 2018). Here, we sought to assay the interaction of RTKN-1 with canonical Rho family members, including RHO-1/RhoA, CDC-42/Cdc42, and CED-10/Rac1. The GST pulldown assay revealed specific binding of RTKN-1 to active RHO-1(GMP-PNP) (Fig. 4 A). We then examined whether the depletion of these GTPases affects the localization of RTKN-1. To circumvent embryonic lethality, synchronized L1 larvae were fed with double-stranded RNA–producing bacteria and scored at the day 1 adult stage (Fig. 4, B–E'). Unexpectedly, the distribution of RTKN-1-GFP was not affected by the loss of these GTPases (Fig. 4, F and F'), implying that RTKN-1 may not act as an effector of the Rho family of GTPases in intestinal cells. Consistently, the localization of RTKN-1-GFP did not show significant variations in cells overexpressing the gain-of-function RHO-1(G14V) or dominant-negative RHO-1(T19N) mutant forms (Fig. 4, F and F').

Rhotekin has been shown to sequester active Rho GTPase (Fu et al., 2000). Also, Rhotekin has been suggested to enhance RhoA activity and stress fiber formation (Pusapati et al., 2012). Per this inference, loss of Rho GTPases should disturb recycling, as observed in RTKN-1–deficient cells. However, there were no distribution irregularities of hTAC-GFP in RHO-1– or CDC-42–depleted cells (Fig. 4, G and G'). Likewise, the subcellular localization of hTAC-GFP was not affected by the overexpression of RHO-1(G14V) or RHO-1(T19N) (Fig. 4, G and G'). Notably, CDC-42 deficiency induced intracellular overaccumulation of hTfR-GFP (Fig. 4, H and H'). Since the loss of CDC-42 failed to disturb the distribution of recycling endosomes (Gao et al., 2020), we speculated that this phenotype could be due to the blockage of ESCRT-mediated degradative traffic (Macara and Spang, 2006; Lu and Drubin, 2020). It should also be noted that CED-10 is capable of recruiting RAB-5-GAP/TBC-2 onto endosomes, thus shutting down RAB-5 activity to facilitate the recycling (Sun et al., 2012). Given the intact localization of RTKN-1-GFP in CED-10–depleted cells (Fig. 4, F and F'), the recycling implication of RTKN-1 is unlikely to be directed by CED-10. Taken together, these results suggested that RTKN-1 functions independently of the Rho family of GTPases to facilitate recycling in the intestine and that Rho/Rhotekin signaling is unlikely involved in this process.

### RTKN-1 impedes UNC-60A/cofilin–mediated actin disassembly

**Figure 5.**
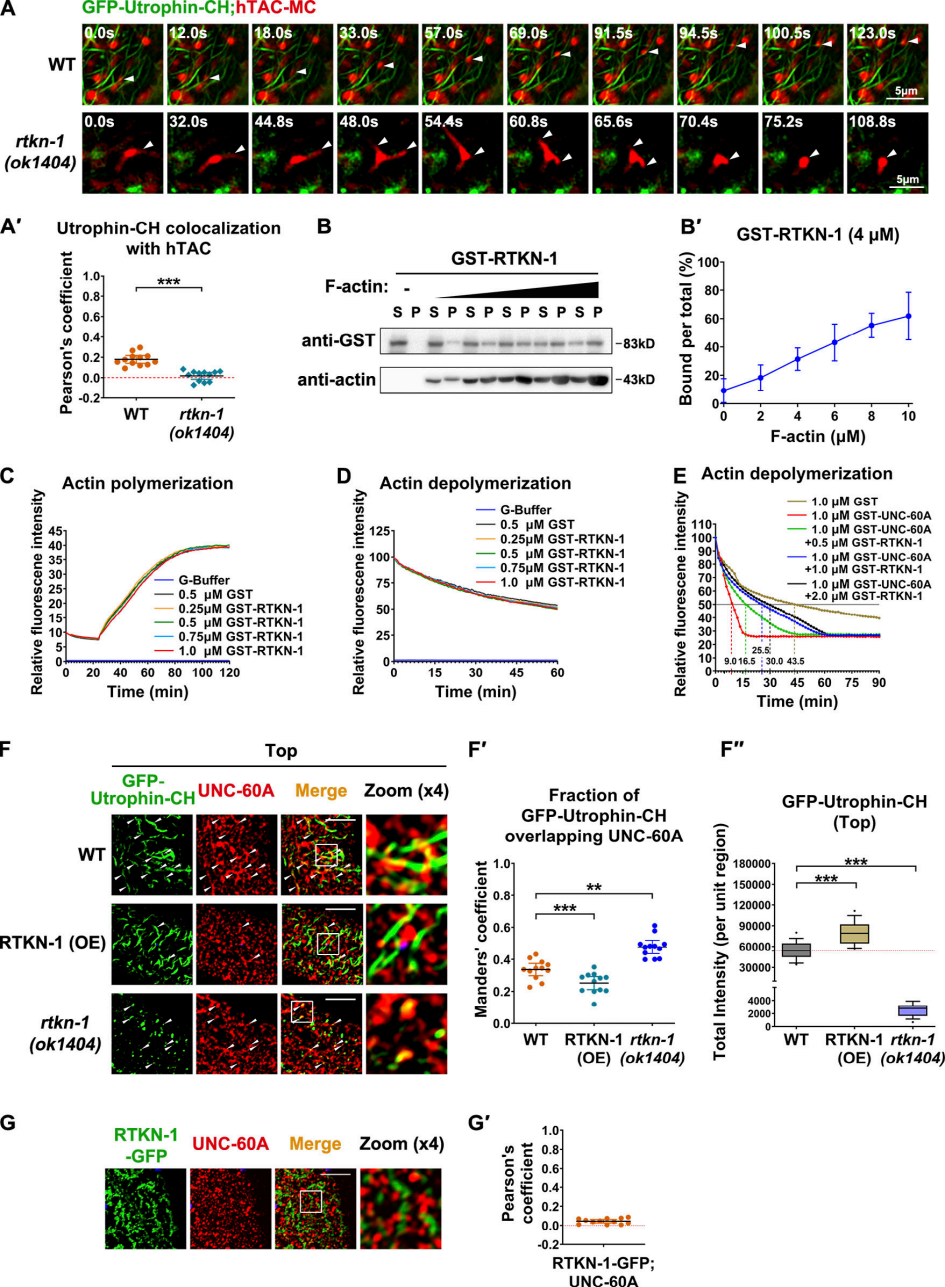
**RTKN-1 impedes UNC-60A–mediated actin disassembly.** **(A and A′)** Live cell fluorescence images showing the dynamic correlation between GFP-Utrophin-CH– and hTAC-GFP–labeled structures. White arrowheads indicate the hTAC-labeled mobile membrane carrier or dynamic tubules. Pearson's correlation coefficients were calculated; error bars are 95% CIs (*n* = 12 animals). ***, P < 0.001 by Mann-Whitney test. **(B and B′)** The actin-binding potential of GST-RTKN-1 was measured using the cosedimentation assay. Multiple concentrations were deployed to make a curve to estimate interaction (*n* = 3 independent experiments). **(C)** Actin polymerization was not affected by the presence of GST-RTKN-1 at various concentrations. **(D)** F-actin depolymerization was not affected by the presence of GST-RTKN-1 at various concentrations. **(E)** GST-RTKN-1 deferred GST-UNC-60A–mediated F-actin depolymerization, *t*_1/2_ has been indicated. **(F and F′)** Confocal images showing the colocalization between endogenous UNC-60A and Utrophin-CH–labeled actin structures. OE, overexpression. Mander's coefficients were calculated; error bars are 95% CIs (*n* = 12 animals). **, P < 0.01; ***, P < 0.001 by Mann-Whitney test. **(F′′)** Fluorescence intensity measurement of GFP-Utrophin-CH. Box-and-whisker plots (*n* = 18 cells): 10–90th percentile; dots, outliers; red midline, median of WT; boundaries, quartiles. ***, P < 0.001 by one-way ANOVA followed by Dunn’s post hoc multiple comparison test. **(G and G′)** Confocal images showing the colocalization between endogenous UNC-60A and RTKN-1-GFP. Pearson's correlation coefficients were calculated; error bars are 95% CIs (*n* = 12 animals). Scale bars, 10 µm. P, pellet; S, supernatant.

Our results introduced RTKN-1 as a novel regulatory protein involved in recycling transport and actin integrity. To directly evaluate the requirement of F-actin for recycling flow in the *C. elegans* intestine, we assayed the dynamic correlation between Utrophin-CH– and hTAC-labeled structures. In a WT background, newly formed hTAC-positive membrane carriers emanated from recycling endosomes, moved along the actin filaments, and finally fused with the opposite endosomal structures (Fig. 5, A and A'; and Video 1). However, in the absence of RTKN-1, some hTAC-positive tubules were still extending but eventually retracted, and Utrophin-CH–labeled actin structures barely overlapped with these membrane tubules (Fig. 5, A and A'; and Video 2). These results corroborated the notion that endosome-associated F-actin is the physical support for the formation, stabilization, and movement of tubular membrane carriers (Simonetti and Cullen, 2019). It is also worth noting that Utrophin-CH preferentially labels Arp2/3-dependent branched F-actin, such as endocytic actin patches and synaptic actin network in *C. elegans* HSN neuron (Chia et al., 2014). Thus, we speculated that the highly dynamic recycling endosome is mainly associated with branched F-actin structures. Indeed, the branched F-actin has been reported to play roles in intracellular membrane dynamics, such as shaping and fission (Picco et al., 2018; Kessels and Qualmann, 2021; Ripoll et al., 2018; Akamatsu et al., 2020).

**Video 1.** **Time-lapse analysis of hTAC-mCherry–labeled endosomal dynamics in WT animal coexpressing GFP-Utrophin-CH.** Young adult animals (24 h after the L4 stage) were used for analysis. Frames were taken every 1.5 s over 123 s. Please refer to the scale bars in Fig. 5 A.

**Video 2.** **Time-lapse analysis of hTAC-mCherry labeled endosomal dynamics in *rtkn-1(ok1404)* mutant animal coexpressing GFP-Utrophin-CH.** Young adult animals (24 h after the L4 stage) were used for analysis. Frames were taken every 1.6 s over 108.8 s. Please refer to the scale bars in Fig. 5 A.

**Figure S4.**
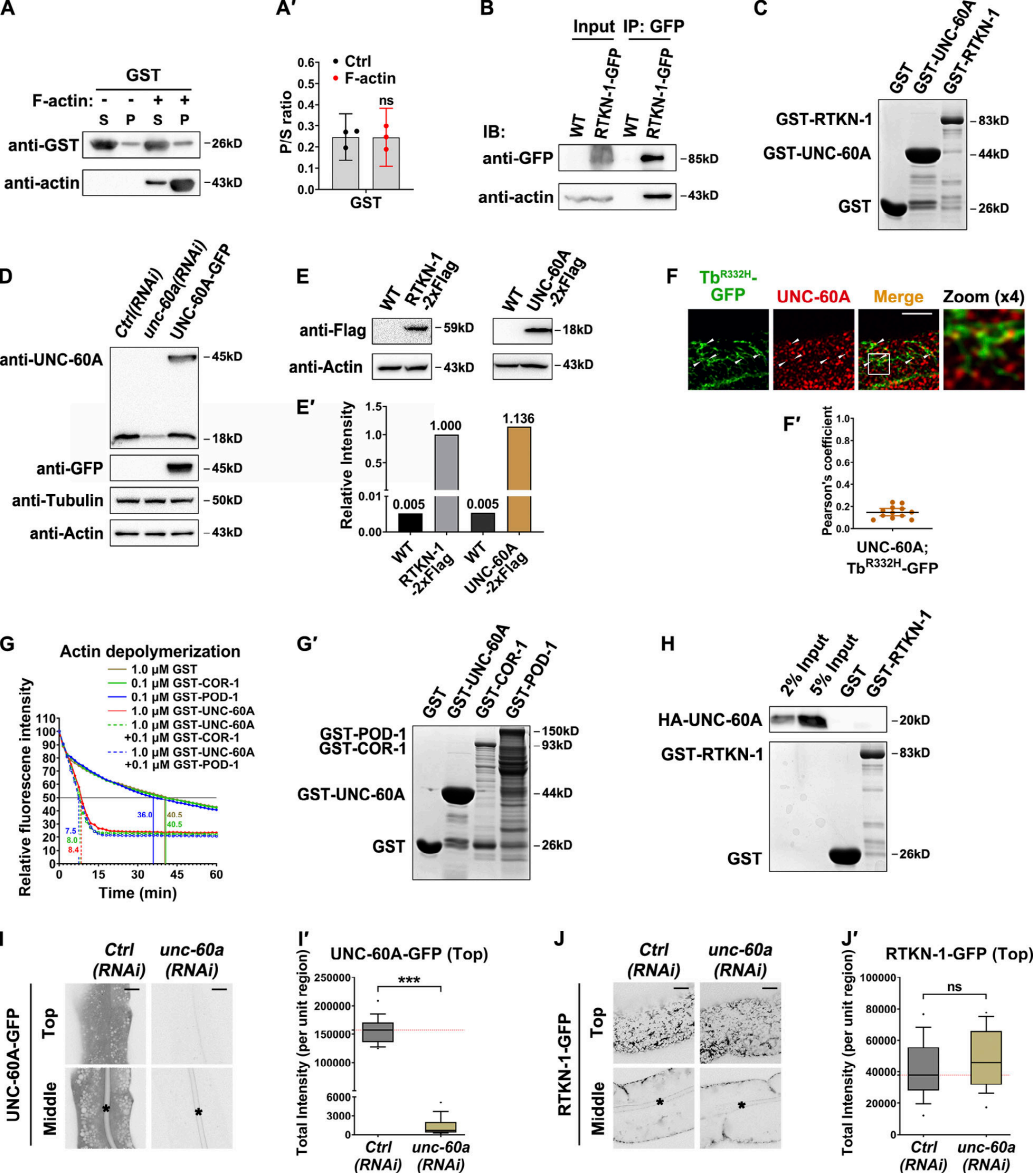
**RTKN-1 interacts with F-actin but not with UNC-60A. (A and A′)** The actin-binding potential of GST was measured using the cosedimentation assay. Band intensity was quantified by using the “Plot Lanes” function of ImageJ; error bars are 95% CIs (*n* = 3 independent experiments). No significant difference by Student’s *t* test. **(B)** Coimmunoprecipitation assay analyzing RTKN-1–actin interaction. **(C)** GST-tagged UNC-60A and RTKN-1 were separated on SDS-PAGE and stained with Coomassie blue. **(D)** Western blot showing UNC-60A (18 kD) and UNC-60A-GFP (45 kD) in WT, *unc-60a(RNAi)*, and UNC-60A-GFP animals. **(E and E′)** The endogenous level of UNC-60A or RTKN-1 (UNC-60A::2xFlag and RTKN-1::2xFlag CRISPR knockin strains). Relative intensity of UNC-60A or RTKN-1 was calculated with reference to the actin level, and the normalized level of RTKN-1 served as a standard (1.000). **(F and F′)** Confocal images showing colocalization between endogenous UNC-60A and PI(4,5)P2 reporter Tubby (Tb)-PH^(R332H)^. The white arrowheads indicate structures labeled by both GFP and mCherry. Pearson's correlation coefficients were calculated; error bars are 95% CIs (*n* = 12 animals). **(G and G′)** The presence of GST-COR-1 or GST-POD-1 did not affect GST-UNC-60A–mediated actin depolymerization. GST-tagged proteins were separated on SDS-PAGE and stained with Coomassie blue. **(H)** Western blot showing GST pulldown with in vitro–translated HA-tagged UNC-60A. There was no interaction between RTKN-1 and UNC-60A. **(I–J′)** Confocal images showing RNAi-mediated knockdown of UNC-60A-GFP and the subcellular distribution of RTKN-1-GFP. Box-and-whisker plots (*n* = 18 cells): 10–90th percentile; dots, outliers; red midline, median of WT; boundaries, quartiles. ***, P < 0.001 by Mann-Whitney test. The black asterisks in the image panels indicate intestinal lumen. Scale bars, 10 µm. Ctrl, control; IB, immunoblot; IP, immunoprecipitation; P, pellet; S, supernatant.

Given the above findings, we probed whether RTKN-1 can interact with F-actin using an F-actin cosedimentation assay. We noticed that the presence of increasing concentrations of F-actin gradually boosted the proportion of GST-RTKN-1 in the precipitate (Fig. 5, B and B'; and Fig. S4, A and A'). We validated the F-actin interaction of RTKN-1 via coimmunoprecipitation (Fig. S4 B). Actin filaments often undergo additional assembly (branching and bundling) and disassembly (severing and depolymerization) to establish a network, fulfilling diverse functions (Paavilainen et al., 2008; Pollard, 2016). The actin structure decrease in *rtkn-1* mutants raised the possibility that RTKN-1 could promote actin polymerization directly. Alternatively, RTKN-1–mediated repression of actin depolymerization can reconcile this phenotype. To distinguish these possibilities, we examined the effects of RTKN-1 on actin in vitro (F-actin 10 µM). The presence of RTKN-1 (0.25–1 µM) had no discernible effect on actin polymerization (Fig. 5 C and Fig. S4 C). Also, RTKN-1 failed to restrain the spontaneous F-actin depolymerization (Fig. 5 D). Considering that PTRN-1/CAMSAPs promote actin polymerization during endocytic recycling (Gong et al., 2018), our data prompted that the positive effect of RTKN-1 on endosomal actin integrity is indirect and that the actin disassembly factor could be the regulatory object of RTKN-1.

Cofilin is a well-appreciated actin regulator that facilitates actin turnover by severing actin filaments (Goode et al., 2001; Kanellos and Frame, 2016). Being homologous proteins of cofilin in *C. elegans*, UNC-60A/F53E2.2 and UNC-60B are generated by alternative splicing of the corresponding transcript (Ono and Ono, 2002; Hagedorn et al., 2014) of which UNC-60A is predominantly expressed in the intestine (Kuroyanagi, 2013). By using the antibody raised against UNC-60A (Fig. S4 D), we found that UNC-60A partially colocalized with Tubby-PH^(R332H)^ on recycling endosomes (Fig. S4, F and F'). A previous study showed that Cor1B/coronin and actin-interacting protein 1 (AIP1) act in concert with Cof1/cofilin to enhance F-actin disassembly (Jansen et al., 2015). In *C. elegans*, AIP1 homologues AIPL-1 and UNC-78 were found to specifically work with the muscle-specific UNC-60B in actin disassembly (Ono et al., 2011). Hence, we focused on coronin homologues COR-1 and POD-1. Unlike UNC-60A, COR-1 or POD-1 did not enhance F-actin depolymerization directly (Fig. S4, G and G'). Also, the UNC-60A–mediated depolymerization was not affected by the presence of GST-COR-1 or GST-POD-1 (Fig. S4, G and G'), indicating that UNC-60A is sufficient to promote actin depolymerization by itself.

Given the actin-binding potential of RTKN-1, we reasoned that RTKN-1 could mitigate the competence of UNC-60A. To test this idea, we first prepared CRISPR-based knockin strains UNC-60A::2xFlag and RTKN-1::2xFlag. Through Western blotting, we estimated that the endogenous RTKN-1/UNC-60A molar ratio is ∼1.136 (Fig. S4 E). Next, we assayed the efficiency of F-actin disassembly accordingly. As expected, UNC-60A (1 µM) accelerated the progression of F-actin (1 µM) depolymerization (Fig. 5 E and Fig. S4 C). In the presence of RTKN-1 (0.5, 1, and 2 µM), the depolymerization was substantially deferred, and this effect was concentration dependent (Fig. 5 E). Regarding this observation, one explanation is that the interaction between RTKN-1 and actin could interfere with the association of UNC-60A with F-actin. UNC-60A was shown to associate with F-actin for a short period, quickly disassemble F-actin, and enter the supernatant (Ono and Benian, 1998). This dynamic allocation precludes us from examining the actin-binding status of UNC-60A in vitro. To evaluate this possibility, we turned to determine whether the residency of UNC-60A in actin structures can be affected by RTKN-1 in vivo. Phalloidin is likely to compete with cofilin for F-actin binding (Nishida et al., 1987), causing false-positive results. Thus, we deployed Utrophin-CH and noticed that although overexpressed RTKN-1 led to an increase of actin structures, the localization of UNC-60A in actin structures was reduced (Fig. 5, F–F"). In the absence of RTKN-1, actin structures near the basolateral membrane were decreased, and UNC-60A could be found on actin remnants (Fig. 5, F and F'). Importantly, we observed no evident overlap between RTKN-1-GFP and UNC-60A (Fig. 5, G and G'). Likewise, we did not identify the binding of RTKN-1 to UNC-60A (Fig. S4 H). Together these findings suggested the existence of an RTKN-1–mediated impediment toward the F-actin association of UNC-60A and that this process does not involve an RTKN-1–UNC-60A interaction.

### Loss of UNC-60A alleviates recycling defects in RTKN-1–depleted cells

**Figure 6.**
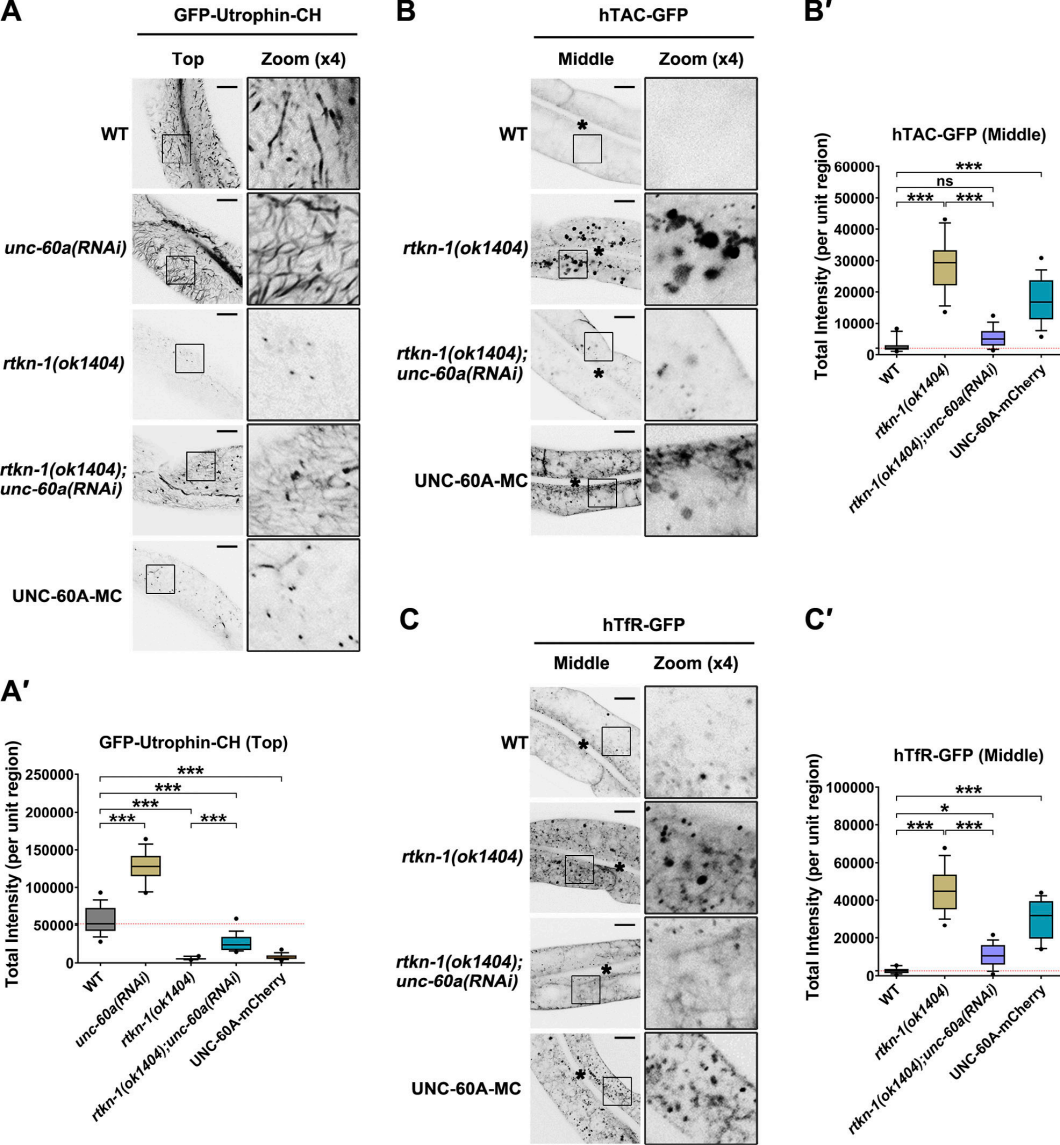
**Loss of UNC-60A mitigates recycling defects in RTKN-1–deficient cells. (A and A′)** Confocal images showing the distribution of Utrophin-CH in intestinal cells. Box-and-whisker plots (*n* = 18 cells): 10–90th percentile; dots, outliers; red midline, median of WT; boundaries, quartiles. ***, P < 0.001 by one-way ANOVA followed by Dunn’s post hoc multiple comparison test. **(B–C′)** Confocal images showing the distribution of hTAC-GFP and hTfR-GFP. Box-and-whisker plots (*n* = 18 cells): 10–90th percentile; dots, outliers; red midline, median of WT; boundaries, quartiles. *, P < 0.05; ***, P < 0.001 by one-way ANOVA followed by Dunn’s post hoc multiple comparison test. The black asterisks in the image panels indicate intestinal lumen. Scale bars, 10 µm.

Our results indicated that RTKN-1 could antagonize UNC-60A–mediated endosomal actin disassembly. To determine the reciprocal influence of UNC-60A on RTKN-1–assisted actin integrity, we assayed the effects of the UNC-60A deficiency in *rtkn-1* mutants. In line with the canonical role of cofilin, actin structures overaccumulated in UNC-60A–depleted cells and diminished upon overexpression of UNC-60A-mCherry (Fig. 6, A and A'), which also indicated that transgenic UNC-60A-mCherry effectively disassembled F-actin in vivo. Of note, in RTKN-1–depleted cells, loss of UNC-60A augmented the level of actin structures (∼38% recovery; Fig. 6, A and A'), suggesting that actin decrease in *rtkn-1* mutants is due at least in part to the enhanced efficacy of UNC-60A. By contrast, the subcellular localization of RTKN-1 was not affected by UNC-60A depletion (Fig. S4, I–J'), implying that the endosomal targeting of RTKN-1 is independent of UNC-60A.

To determine whether RTKN-1 deficiency–induced elevation of UNC-60A potency is implicated in the recycling defects, we examined the distribution of recycling cargos in *rtkn-1;unc-60a* double knockdown animals. In agreement with the variation of actin structures, the overaccumulation of hTAC-GFP or hTfR-GFP in RTKN-1–deficient cells was mitigated by the concomitant loss of UNC-60A (Fig. 6, B–C'). Further highlighting the significance of actin integrity in recycling transport, ectopic expression of UNC-60A-mCherry led to recycling cargo overaccumulation in the cytosol (Fig. 6, B–C').

### RBD and PH-CT are required for the functionality of RTKN-1

**Figure 7.**
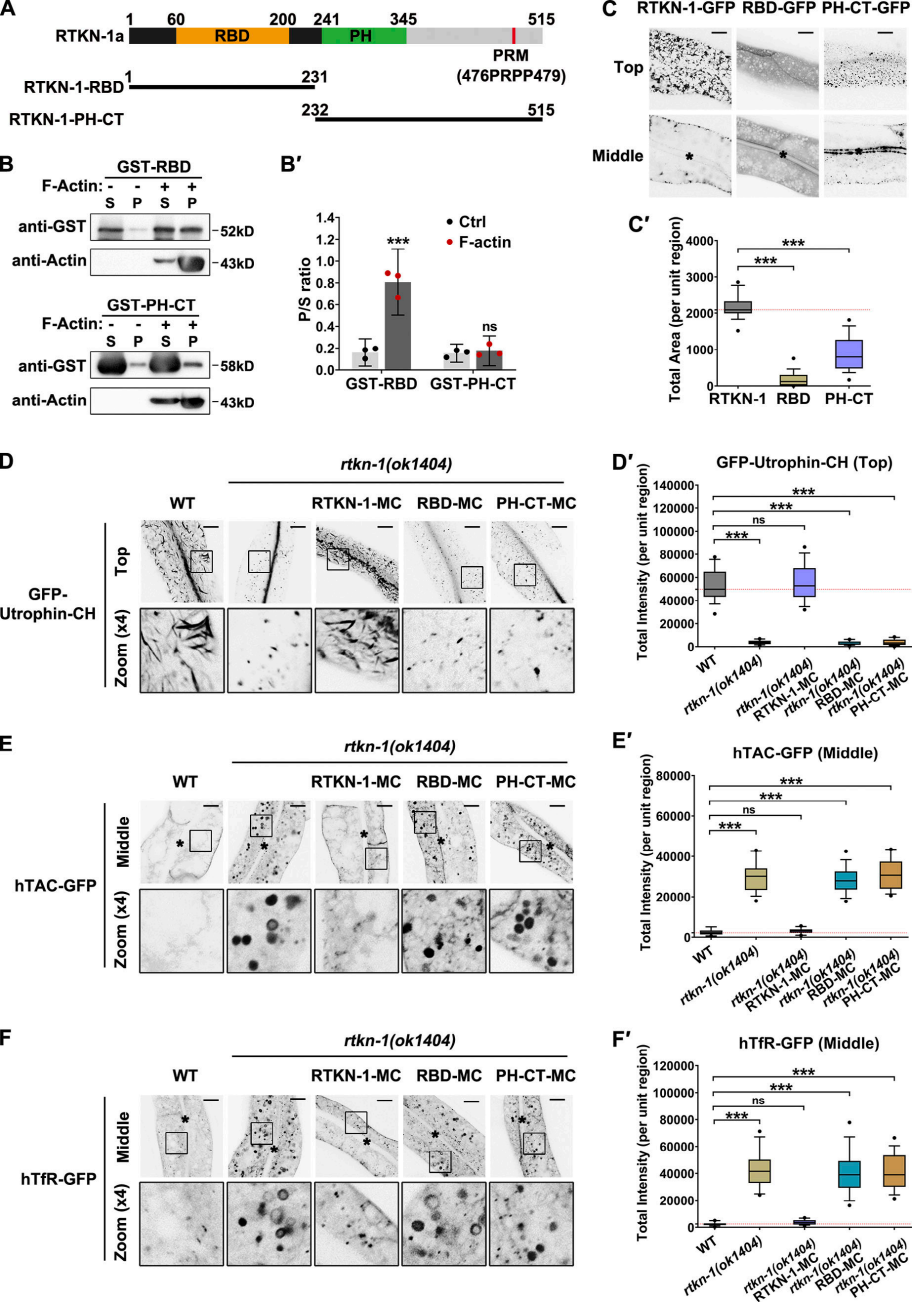
**N-terminal RBD and PH-CT are required for RTKN-1 functionality. (A)** Domain architecture of RTKN-1a and fragments. **(B and B′)** The actin-binding potential of the purified GST-RBD or GST-PH-CT was measured by using the cosedimentation assay. Band intensity was quantified by using the “Plot Lanes” function of ImageJ; error bars are 95% CIs (*n* = 3 independent experiments). ***, P < 0.001 by Student’s *t* test. **(C and C′)** Confocal images showing subcellular localization of RTKN-1-GFP, RBD-GFP, and PH-CT-GFP in intestinal cells. Box-and-whisker plots (*n* = 18 cells): 10–90th percentile; dots, outliers; red midline, median of WT; boundaries, quartiles. ***, P < 0.001 by one-way ANOVA followed by Dunn’s post hoc multiple comparison test. **(D–F′)** Confocal images showing the distribution of GFP-Utrophin-CH, hTAC-GFP, and hTfR-GFP in intestinal cells. Box-and-whisker plots (*n* = 18 cells): 10–90th percentile; dots, outliers; red midline, median of WT; boundaries, quartiles. ***, P < 0.001 by one-way ANOVA followed by Dunn’s post hoc multiple comparison test. The black asterisks in the image panels indicate intestinal lumen. Scale bars, 10 µm. Ctrl, control; MC, mCherry; P, pellet; S, supernatant.

RTKN-1 contains an RBD, a PH domain, and a C-terminus bearing a PRM. RBD mediates interactions with the Rho family of GTPases (Dvorsky et al., 2004; Ito et al., 2018), while the PH domain often presents a phospholipid affinity (Someya et al., 2001; Xu et al., 2018). We split RTKN-1 into an RBD segment and a portion comprising the PH-CT (Fig. 7 A). We observed that RBD still interacted with F-actin (Fig. 7, B and B'). Live cell imaging showed that RBD-GFP appeared diffusively in the cytosol (Fig. 7, C and C'). Surprisingly, PH-CT-GFP did not display a meshwork pattern and often resided in the intracellular puncta (Fig. 7, C and C'), suggesting that the RBD-actin interaction contributes to RTKN-1 localization and/or maintenance of recycling endosomal architecture.

We then probed the functional relevance of RBD and PH-CT. As expected, the expression of PH-CT-mCherry failed to relieve the actin diminishment phenotype in RTKN-1–depleted cells (Fig. 7, D and D'). Unexpectedly, the presence of RBD did not restore the level of actin structures (Fig. 7, D and D'), indicating that the RTKN-1–assisted actin integrity requires the synergism of RBD and the downstream region. Consistently, overexpression of RBD-mCherry or PH-CT-mCherry did not complement the recycling defects (Fig. 7, E–F'), establishing the implication of both RBD and PH-CT in recycling modulation.

### CT is imperative for RTKN-1 oligomerization

**Figure 8.**
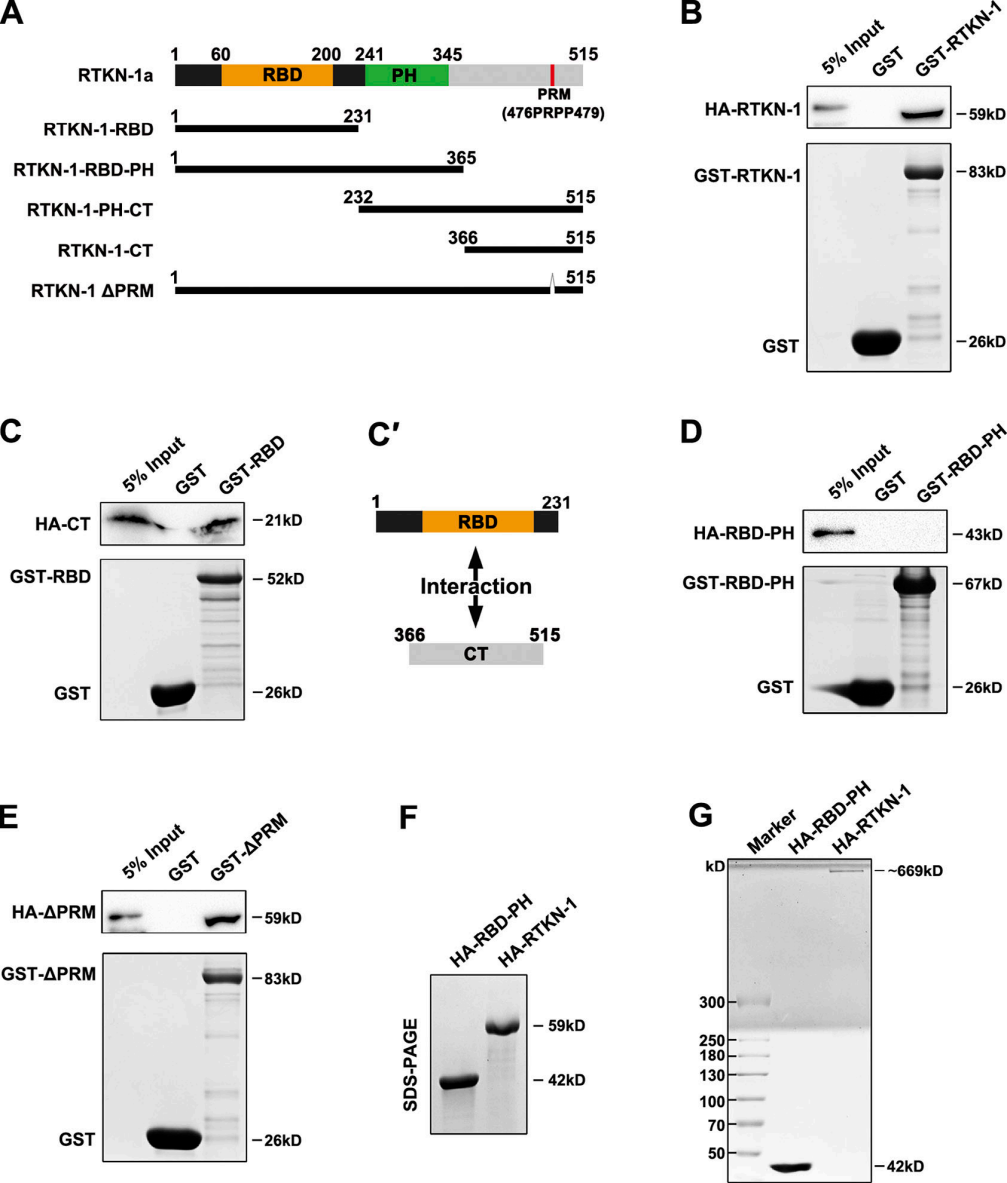
**RTKN-1-CT is imperative for RTKN-1 oligomerization. (A)** Domain architecture of RTKN-1a and fragments, including RBD, RBD-PH, PH-CT, CT, and ΔPRM. Δ indicates deletion. **(B)** Western blot showing GST pulldown with in vitro–translated HA-tagged RTKN-1. **(C and C′)** Western blot showing GST pulldown with in vitro–translated HA-tagged RTKN-1-CT. **(D)** Western blot showing GST pulldown with in vitro–translated HA-RBD-PH. **(E)** Western blot showing GST pulldown with in vitro translated HA-RTKN-1 ΔPRM. **(F)** SDS-PAGE of purified HA-RBD-PH and HA-RTKN-1. **(G)** Gradient SDS-PAGE of covalently crosslinked HA-RBD-PH and HA-RTKN-1.

**Figure S5.**
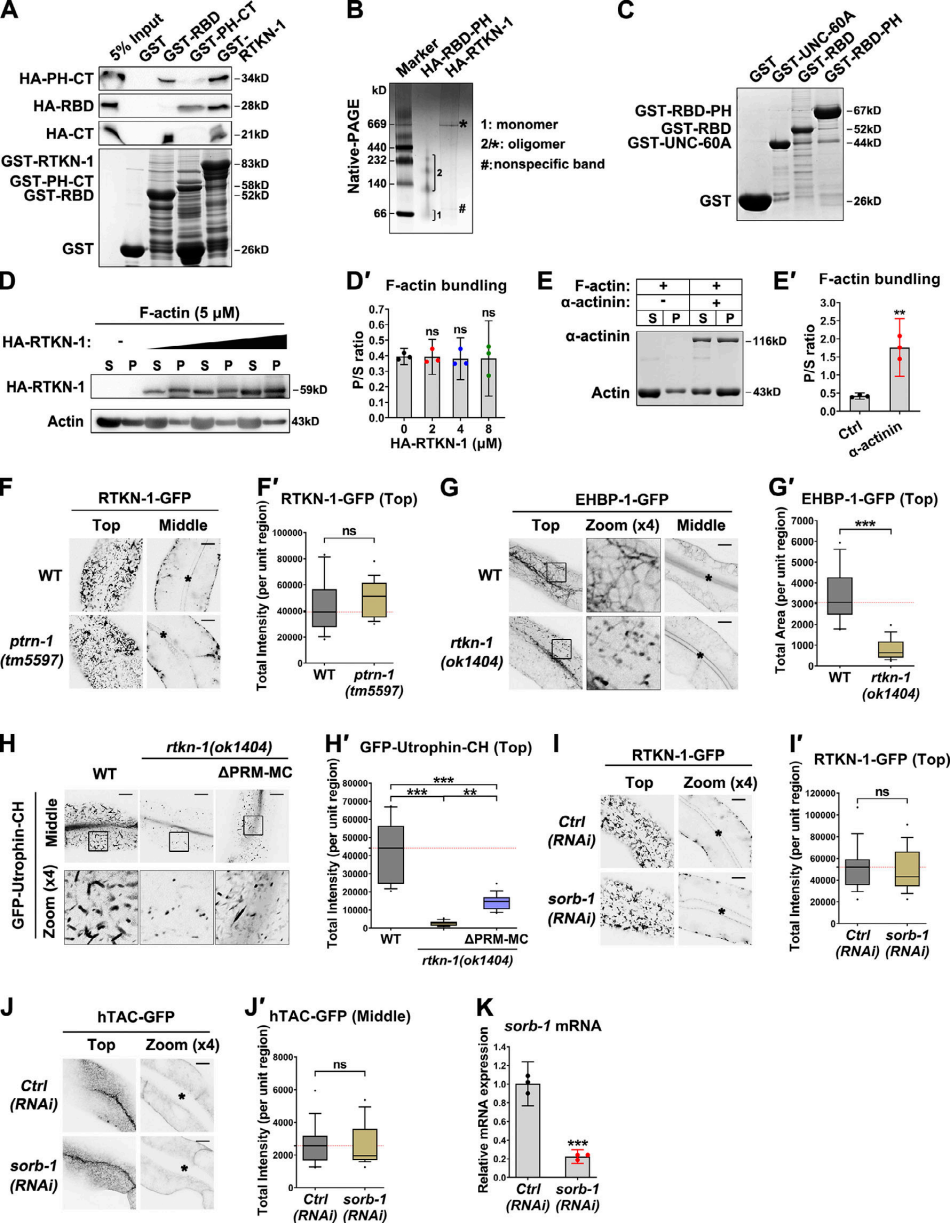
**CT behind PH domain mediates RTKN-1 self-association and RTKN-1 functions independently of PTRN-1 and SORB-1 in the intestine.** **(A)** Western blot showing GST pulldown with in vitro–translated HA-tagged proteins. GST-RBD exhibited an interaction with HA-CT. **(B)** Native PAGE of uncrosslinked HA-RBD-PH and HA-RTKN-1. **(C)** GST-tagged proteins were separated on SDS-PAGE and stained with Coomassie blue. **(D–E′)** The actin-bundling capacity of HA-RTKN-1 was measured using the cosedimentation assay. α-Actinin was used as a positive control. Multiple concentrations were deployed to estimate the pellet/supernatant (P/S) ratio. Error bars are 95% CIs (*n* = 3 independent experiments). **, P < 0.01 by Student’s *t* test. **(F–H′)** Confocal images showing the subcellular localization of RTKN-1-GFP, EHBP-1-GFP, and GFP-Utrophin-CH in intestinal cells. Box-and-whisker plots (*n* = 18 cells): 10–90th percentile; dots, outliers; red midline, median of WT; boundaries, quartiles. **, P < 0.01; ***, P < 0.001 by Mann-Whitney test. **(I–J′)** The subcellular localization of RTKN-1-GFP or hTAC-GFP was not significantly affected in *sorb-1(RNAi)* cells. Box-and-whisker plots (*n* = 18 cells): 10–90th percentile; dots, outliers; red midline, median of WT; boundaries, quartiles. No significant difference by Mann-Whitney test. **(K)** mRNA level showing RNAi-mediated knockdown efficiency of SORB-1. Error bars are 95% CIs (*n* = 3 independent experiments). ***, P < 0.001 by Student’s *t* test. The black asterisks in the image panels indicate intestinal lumen. Scale bars, 10 µm. Ctrl, control; MC, mCherry.

Tropomyosin can be joined end to end to form a cable that extends along the sides of the actin filaments (Christensen et al., 2017; Skoumpla et al., 2007), thus competing with cofilin for actin association (Kuhn and Bamburg, 2008; Ono and Ono, 2002). Our evidence suggested that RTKN-1 could interfere with the association of UNC-60A with endosomal actin structures. An intriguing scheme arises as to whether RTKN-1 is capable of self-associating and thus shields F-actin from cofilin. To test this idea, we first identified the self-binding capacity of RTKN-1 (Fig. 8, A and B), which is mediated by the RBD and CT beyond the PH domain (Fig. 8, C and C'; and Fig. S5 A). Accordingly, the RTKN-1 fragment lacking the CT (RBD-PH) lost the self-binding potential (Fig. 8 D). Of note, the C-terminal PRM is not required for this self-association (Fig. 8 E). Next, we determined whether RTKN-1 could form oligomers via this RBD-CT interaction. In SDS-PAGE gel, purified HA-RTKN-1 yielded a band around 59 kD (Fig. 8 F). However, after protein was covalently crosslinked, the HA-RTKN-1 band shifted to a high molecular mass range, and a prominent band of ∼669 kD appeared in the gel (Fig. 8 G). In contrast, HA-RBD-PH remained in the form of the monomer (Fig. 8 G). Likewise, uncrosslinked HA-RTKN-1 presented a band of ∼669 kD in the native PAGE gel (Fig. S5 B). Together these results suggested that RTKN-1 could form an oligomer, and CT is indispensable for this assembly.

Our previous study showed that actin-bundling protein EHBP-1 could assist in recycling endosomal tubulation (Wang et al., 2016). Nonetheless, by implementing a low-speed cosedimentation assay, RTKN-1 did not show actin-bundling potency (Fig. S5, D–E'), suggesting that oligomerization does not endow RTKN-1 with the ability to promote actin bundling. Recently, PTRN-1/CAMSAP was found to promote actin polymerization during endocytic recycling (Gong et al., 2018). In the absence of PTRN-1, the distribution of RTKN-1-GFP was intact (Fig. S5, F and F'), indicating that the subcellular localization of RTKN-1 does not require PTRN-1. Indeed, these results are consistent with the identification of PTRN-1 as a resident protein in punctate early/sorting endosomes, modulating actin network architecture there.

### CT is a determinant for actin integrity during basolateral recycling

**Figure 9.**
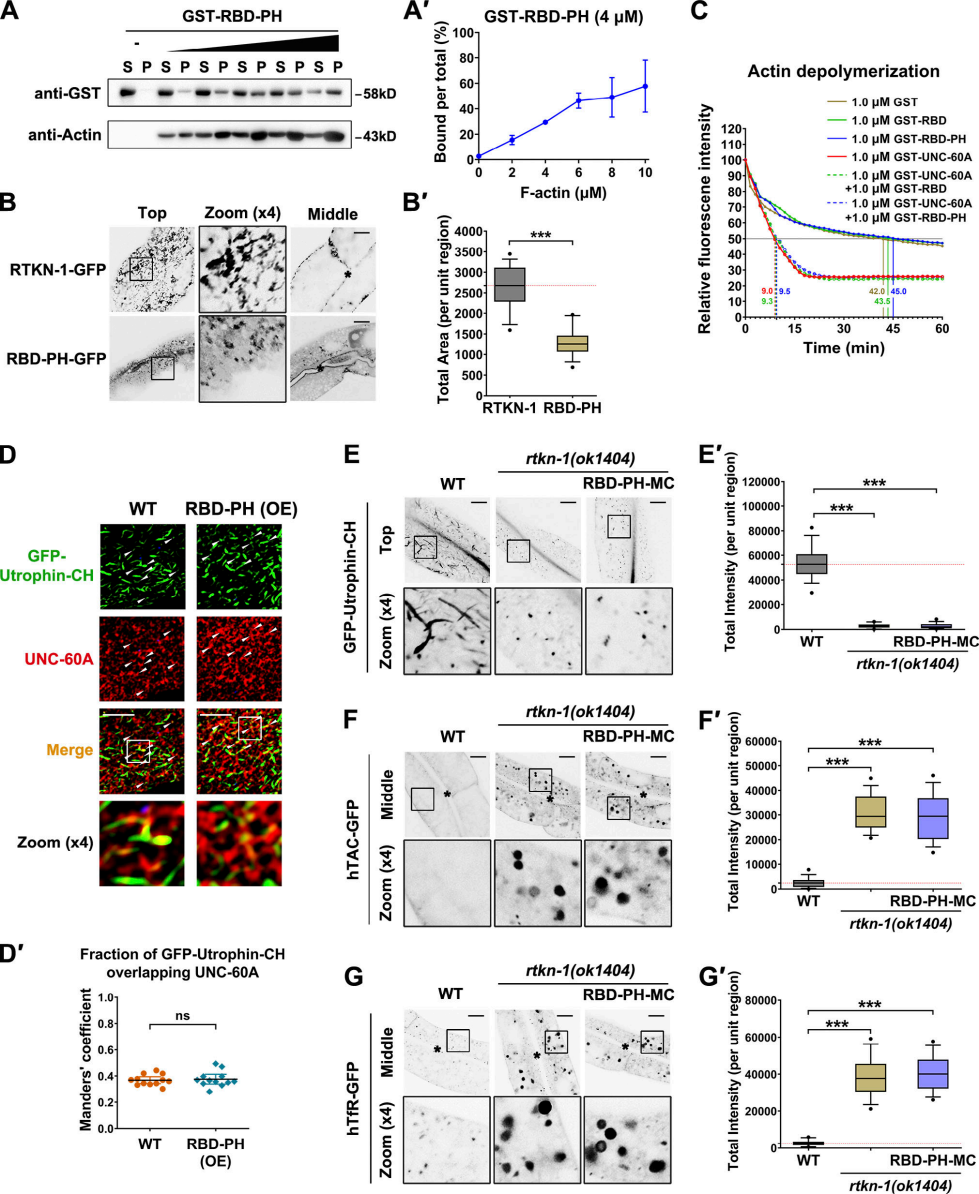
**RTKN-1-CT is a determinant for actin integrity during basolateral recycling. (A and A′)** The actin-binding potential of the GST-RBD-PH was measured using the cosedimentation assay. Multiple concentrations were deployed to make a curve to estimate interaction (*n* = 3 independent experiments). **(B and B′)** Confocal images showing localization of RTKN-1-GFP and RBD-PH-GFP in intestinal cells. Box-and-whisker plots (*n* = 18 cells): 10–90th percentile; dots, outliers; red midline, median of WT; boundaries, quartiles. ***, P < 0.001 by Mann-Whitney test. **(C)** GST-RBD or GST-RBD-PH did not affect GST-UNC-60A–mediated actin depolymerization; t_1/2_ has been indicated. **(D and D′)** Confocal images showing the colocalization between endogenous UNC-60A and Utrophin-CH–labeled actin structures. The white arrowheads indicate structures labeled by both GFP and mCherry. OE, overexpression. Mander's coefficients were calculated; error bars are 95% CIs (*n* = 12 animals). **(E–G′)** Confocal images showing the distribution of GFP-Utrophin-CH, hTAC-GFP, and hTfR-GFP in intestinal cells. Box-and-whisker plots (*n* = 18 cells): 10–90th percentile; dots, outliers; red midline, median of WT; boundaries, quartiles. ***, P < 0.001 by one-way ANOVA followed by Dunn’s post hoc multiple comparison test. The black asterisks in the image panels indicate intestinal lumen. Scale bars, 10 µm. MC, mCherry; P, pellet; S, supernatant.

To explore the significance of the C-terminus of RTKN-1, we first analyzed the actin-binding capability of the RBD-PH. As expected, RBD-PH maintains an interaction with F-actin (Fig. 9, A and A'). However, RBD-PH-GFP only exhibited punctate labeling (Fig. 9, B and B'), suggesting that the CT is essential for the generation and/or maintenance of recycling endosomal architecture. Consistently, the recycling endosomal meshwork labeled by EHBP-1-GFP collapsed in *rtkn-1* mutants (Fig. S5, G and G'). We next assessed whether CT is required for actin integrity. Remarkably, the UNC-60A–assisted actin depolymerization was not affected by the presence of RBD or RBD-PH (Fig. 9 C and Fig. S5 C), indicating that RBD–actin interaction is not sufficient to defer actin disassembly. After that, we assayed the effect of RBD-PH overexpression on colocalization between UNC-60A and actin structures. The overlap between UNC-60A and actin was not significantly affected (Fig. 9, D and D'). Similar to the *rtkn-1* mutants, RTKN-1–depleted cells coexpressing RBD-PH-mCherry exhibited a distinguished decrease in Utrophin-CH–labeled actin structures (Fig. 9, E and E'). Also, the overexpression of RBD-PH-mCherry failed to alleviate the overaccumulation of recycling cargos in *rtkn-1* mutants (Fig. 9, F–G'). Taken together, these results highlighted the importance of the RTKN-1 C-terminus as an actin integrity determinant during recycling transport.

### SDPN-1 acts to direct the proper residency of RTKN-1 in recycling endosomes

**Figure 10.**
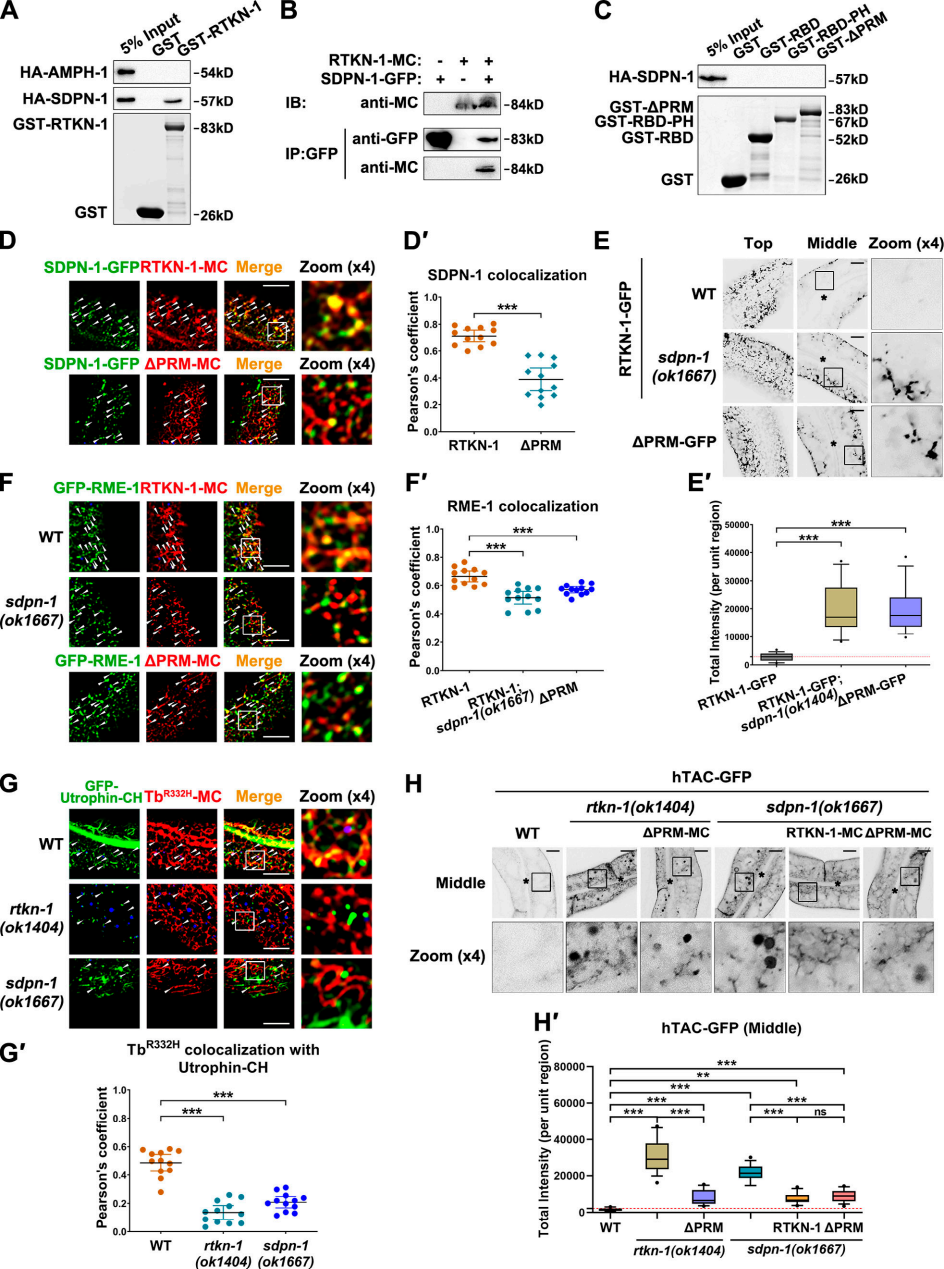
**SDPN-1 directs the localization of RTKN-1 in recycling endosomes. (A)** Western blot showing GST pulldown with in vitro–translated HA-tagged AMPH-1 and SDPN-1. **(B)** Coimmunoprecipitation assay analyzing RTKN-1–SDPN-1 interaction. **(C)** Western blot showing GST pulldown with in vitro–translated HA-SDPN-1. **(D and D′)** Confocal image showing colocalization between SDPN-1-GFP and mCherry-tagged RTKN-1 or RTKN-1ΔPRM in the intestinal cells. The white arrowheads indicate structures labeled by both GFP and mCherry. Pearson’s correlation coefficients for GFP and mCherry signals are calculated; error bar is 95% CI (*n* = 12 animals). ***, P < 0.001 by Mann-Whitney test. **(E and E′)** Confocal images showing the distribution of RTKN-1-GFP. Box-and-whisker plots (*n* = 18 cells): 10–90th percentile; dots, outliers; red midline, median of WT; boundaries, quartiles. ***, P < 0.001 by one-way ANOVA followed by Dunn’s post hoc multiple comparison test. **(F and F′)** Confocal image showing colocalization between GFP-RME-1 and mCherry-tagged RTKN-1 or RTKN-1ΔPRM in the intestinal cells. Pearson’s correlation coefficients for GFP and mCherry signals are calculated; error bar is 95% CI (*n* = 12 animals). ***, P < 0.001 by Mann-Whitney test. **(G and G′)** Confocal image showing colocalization between GFP-Utrophin-CH and Tubby (Tb)-PH^(R332H)^-mCherry in the intestinal cells. Pearson’s correlation coefficients for GFP and mCherry signals are calculated; error bar is 95% CI (*n* = 12 animals). ***, P < 0.001 by Mann-Whitney test. **(H and H′)** Confocal images showing the distribution of hTAC-GFP. Box-and-whisker plots (*n* = 18 cells): 10–90th percentile; dots, outliers; red midline, median of WT; boundaries, quartiles. **, P < 0.01; ***, P < 0.001 by one-way ANOVA followed by Dunn’s post hoc multiple comparison test. The black asterisks in the image panels indicate intestinal lumen. Scale bars, 10 µm. IB, immunoblot; IP, immunoprecipitation; MC, mCherry.

This study noticed that RTKN-1 colocalizes well with the recycling endosomal actin regulators SDPN-1 and EHBP-1, whereas it overlaps with a recycling endosome-associated dynamin-like protein RME-1 to a lesser extent. This distinct phenotype prompts a correlation between RTKN-1 and specific recycling regulators. It is noteworthy that the SH3 domain has been identified as a PRM binding partner (Mayer, 2001; Lim et al., 1994). Therefore, we scanned the sequences of the major recycling regulators and found that SDPN-1/Syndapin and AMPH-1/Amphiphysin carry an SH3 domain. We tested the interaction and observed that RTKN-1 solely binds to SDPN-1 (Fig. 10, A and B). Further analyses revealed the necessity of PRM in the RTKN-1–SDPN-1 interaction (Fig. 10 C).

Syndapin family proteins belong to F-BAR domain membrane-bending proteins and are associated with Arp2/3-dependent actin assembly (Qualmann and Kelly, 2000; Qualmann et al., 1999). Syndapin 2 has been implicated in endosomal function during recycling transport (Braun et al., 2005; Giridharan et al., 2013). Likewise, in *C. elegans* intestinal epithelium, SDPN-1/Syndapin is located in the basolateral recycling endosomes. Loss of SDPN-1 reduced Lifeact labeling on endosomes (Gleason et al., 2016), suggesting that SDPN-1 promotes recycling via actin polymerization or recruitment. In this regard, we explored the mode in which SDPN-1 affects the efficacy of RTKN-1. In agreement with the biochemical results, RTKN-1-mCherry colocalized well with SDPN-1-GFP in the intestinal cells (Fig. 10, D and D'), and this overlap was impaired by loss of PRM (Fig. 10, D and D'). Remarkably, the distribution of RTKN-1-GFP was abnormal in cells lacking SDPN-1, and many RTKN-1-GFP–labeled structures appeared in the deep cytosol (Fig. 10, E and E'). Consistently, although RTKN-1-mCherry was still located in tubular and punctate structures, SDPN-1 deficiency led to a decrease in the overlap between RTKN-1-mCherry and GFP-RME-1 (Fig. 10, F and F'). Similarly, a considerable portion of RTKN-1ΔPRM-mCherry signals was not found on the GFP-RME-1–labeled structures (Fig. 10, F and F'). Taken together, these findings suggested that PRM-mediated interaction with SDPN-1 facilitates the appropriate sequestering of RTKN-1 on recycling endosomes.

To further elaborate on the functional significance of this interaction, we examined the distribution of actin structures in RTKN-1– or SDPN-1–deficient cells. As expected, the residence of GFP-Utrophin-CH–labeled F-actin on Tubby-PH^(R332H)^–positive recycling endosomes was reduced by the loss of RTKN-1 or SDPN-1 (Fig. 10, G and G'). Consistent with this observation, although RTKN-1ΔPRM overexpression could somewhat alleviate the actin diminishment in *rtkn-1* mutants (Fig. S5, H and H'), it was not sufficient to fully rescue the hTAC-GFP recycling defects (Fig. 10, H and H'). Similarly, overexpression of RTKN-1 or RTKN-1ΔPRM could not effectively relieve the overaccumulation of hTAC-GFP in *sdpn-1* mutant animals (Fig. 10, H and H'). Altogether, these results suggested that SDPN-1 acts upstream to direct the proper association of RTKN-1 with recycling endosomes and promote the integrity of endosome-associated actin structures.

## Discussion

Here, we identified RTKN-1/Rhotekin as a requisite for the basolateral recycling in the intestine. Particularly, RTKN-1 mitigated the efficacy of the actin disassembly factor UNC-60A/cofilin. Further analyses suggested that SDPN-1 acts to sequester RTKN-1 onto recycling endosomes, thereby stabilizing endosome-associated F-actin and facilitating membrane carrier formation and movement.

The Rho GTPases have been involved in regulating the actin architecture (Lee and Dominguez, 2010; Camera et al., 2003; Leung et al., 1996; Mott et al., 1999; Bishop and Hall, 2000). Cdc42 exposes the binding surface within N-WASP and activates branched actin nucleator Arp2/3 (Rohatgi et al., 2000). Through binding to mDia/formin GTPase binding domain, RhoA disrupts the self-inhibiting conformation of mDia (Higashida et al., 2004). In addition, RhoA promotes phosphorylation of LIMKs via effector ROCK. Active LIMKs further phosphorylate cofilin, thus inhibiting F-actin disassembly (Geneste et al., 2002). Notably, RhoA effector Rhotekin was observed to affect the activity of RhoA via a feedback mechanism. The overexpression of an S435E Rhotekin caused an increase in active RhoA level and the stress fiber formation (Pusapati et al., 2012). We validated the interaction of RTKN-1/Rhotekin with active RHO-1/RhoA. However, basolateral recycling was not impaired by loss of RHO-1, suggesting that RHO-1 does not act as a regulator of RTKN-1 during endocytic recycling in the intestine. Furthermore, unlike the way in which ROCK directs cofilin activity via phosphorylation (Geneste et al., 2002), RTKN-1 could attenuate the potency of UNC-60A/cofilin by impeding the F-actin association of UNC-60A.

Coronin promotes the association of cofilin with F-actin followed by recruitment of AIP1, which synergistically acts on F-actin for rapid depolymerization (Kueh et al., 2008; Ishikawa-Ankerhold et al., 2010). UNC-78 and AIPL-1 are homologues of AIP1 in *C. elegans*, both of which were perceived to function together with UNC-60B/cofilin and have no engagement with the UNC-60A/cofilin (Mohri and Ono, 2003; Ono and Ono, 2014). The worm homologues of coronin, COR-1 and POD-1, are predicted to be expressed in the intestine (WormBase version WS241). We thus examined the effect of COR-1 and POD-1 on actin depolymerization. Nonetheless, UNC-60A–mediated actin disassembly was not affected by COR-1 or POD-1. Given that UNC-60B was found to be solely expressed in muscle (Ono and Ono, 2002), these findings suggested that although coronin could synergize with AIP1 and UNC-60B in specific tissues or biological processes, the acting scenario of UNC-60A in intestinal epithelia appears to be quite different.

Our analysis prompts that RTKN-1 could adopt two distinct conformations, one of which is a self-inhibiting configuration. Given the distinct interacting partners of the RBD and PH domain (F-actin and membrane, respectively), the self-inhibiting configuration would hinder the interaction of RTKN-1 with the corresponding partners. An alternative conformation could be the formation of oligomers, which could extend on F-actin, thereby limiting the presence of cofilin in actin structures. Further biochemical and structural analysis will be required to justify this interpretation. Regarding the positioning mechanism of RTKN-1/Rhotekin, previous studies showed that the midbody residency of Rhotekin during cell division is governed by vinexin (Chang and Huang, 2017). Vinexin belongs to the sorbin adaptor family, and *C. elegans* SORB-1 is most homologous to vinexin (Loveless et al., 2017). Nevertheless, in *sorb-1(RNAi)* animals, the distribution of RTKN-1-GFP or hTAC-GFP was not affected (Fig. S5, I–K), suggesting that the regulatory role of RTKN-1 during recycling is likely independent of SORB-1. Indeed, we found that the recycling endosomal residence of RTKN-1 requires the presence of SDPN-1.

Utrophin-CH preferentially labels Arp2/3-dependent branched F-actin (Chia et al., 2014). Given the reduced endosomal labeling of the Utrophin-CH in *sdpn-1* mutants (Fig. 10, G and G'), we speculated that the recycling endosome is mainly associated with branched F-actin. Indeed, SDPN-1/Syndapin has been shown to regulate recycling by promoting the formation of a branched F-actin network (Giridharan et al., 2013; Sherlekar et al., 2020; Gleason et al., 2016). Moreover, while demonstrating the functional context of RTKN-1–mediated actin integrity modulation, our data further suggested that SDPN-1 could act as an endosomal actin organization coordinator, which orchestrates assembly and stabilization of the endosome-associated actin and thus promotes the formation and directional movement of recycling membrane carriers. Thus, in addition to previously described endosomal actin dynamics (Simonetti and Cullen, 2019, our study provides new insight into actin organization regulation during endocytic recycling.

## Materials and methods

### General methods and strains

The *C. elegans* strains were cultured at 20°C on nematode growth medium plates with *Escherichia coli* strain OP50 as the food source. RNAi-mediated interference was performed by feeding the *C. elegans* strains with *E. coli* strain HT115 carrying the L4440 vector expressing double-stranded RNA (Timmons and Fire, 1998). If not specifically indicated in the RNAi experiments, animals in the adult stage were cultured, and the next generation was scored at the day 1 adult stage.

### Two-step genome-wide RNAi genetic screen

The* rab-10* mutant is superficially normal in growth and development (Shi et al., 2010; Wang et al., 2016). However, *rab-10* mutants accumulate recycling cargo hTAC-GFP in intestinal cells (Chen et al., 2006). To identify recycling regulators, a genome-wide RNAi screen (19,763 genes) was performed using *rab-10(RNAi)* as the positive control. After the primary screen, the knockdown of 54 candidates was found to lead to intracellular hTAC-GFP aggregation, including RTKN-1. A small-scale screen for actin dynamics regulators was then conducted within these candidates, and loss of RTKN-1 exhibited an adverse effect on GFP-Utrophin-CH–labeled actin structures.

### Antibodies

The rabbit polyclonal antibody was raised against full-length UNC-60A (YouLong Biotech; Fig. S4 D). Other antibodies used in this study include mouse anti-actin monoclonal antibody (sc-47778; Santa Cruz Biotechnology), mouse anti-Flag monoclonal antibody (F1804; Sigma), rabbit anti-HA monoclonal antibody (C29F4; Cell Signaling Technology), mouse anti-GFP monoclonal antibody (AG281; Beyotime), mouse anti-His monoclonal antibody (AH367; Beyotime), and rabbit anti-GST monoclonal antibody (91G1; Cell Signaling Technology).

### Plasmids and transgenic strains

To construct transgenes expressed in the intestine, *vha-6* promoter-driven destination vectors with a Gateway cassette inserted upstream or downstream of the GFP or mCherry coding region were used (Chen et al., 2006). The cDNAs of *rtkn-1a*, *rtkn-1a(1-231aa)*, *rtkn-1a(232-515aa)*, *rtkn-1a(1-365aa)*, *rtkn-1a(366-515aa)*, *unc-60A*, *cor-1*, and *pod-1* were inserted in frame into entry vector pDONR221 by BP reaction. LR reactions were conducted to transfer the coding sequences into destination vectors. The transgenic lines were obtained using the standard microinjection technique. Low-copy transgenic strains were obtained by the microparticle bombardment method (Praitis et al., 2001). For *unc-60a* and *sorb-1* RNAi knockdown, the cDNAs were inserted into a linearized L4440 vector using the ClonExpress II One-Step Cloning Kit (Vazyme).

### CRISPR-Cas9 knockin strains

To generate RTKN-1::2xFlag and UNC-60A::2xFlag knockin alleles, PCR products with homology arms (500 bp upstream and downstream of the guide cut site) were subcloned into a pUC19 vector. The 2xFlag sequence was then inserted before the stop codon of *rtkn-1* or *unc-60a* using overlapping PCR, and the plasmids were used as repair templates. Single gRNA (sgRNA) template containing T7 promoter (5′-AAG​CTA​ATA​CGA​CTC​ACT​ATA​GG-3′), sgRNA sequence (*rtkn-1* sgRNA: 5′-GGG​TGA​CAT​GCG​ATG​CAT​TA-3′; *unc-60a* sgRNA: 5′-AAC​AAG​TTG​GGC​GAG​AAA​TA-3′), and trans-activating CRISPR RNA sequence (5′-GTT​TTA​GAG​CTA​GAA​ATA​GCA​AGT​TAA​AAT​AAG​GCT​AGT​CCG​TTA​TCA​ACT​TGA​AAA​AGT​GGC​ACC​GAG​TCG​GTG​CTT​TT-3′) was prepared using PCR. The PCR products were used as the template for sgRNA transcription using the HiScribe Quick T7 High Yield RNA Synthesis Kit (E2050; New England Biolabs). sgRNA was purified using Monarch RNA Cleanup Kit (T2040; New England Biolabs). sgRNA and 0.5 µl Cas9 nuclease (10 µg/µl; Integrated DNA Technologies) were incubated at 37°C for 10 min, and repair template pUC19 vector (final 200 ng/µl) and selection marker pRF4[*rol-6(su1006)*] vector (final 50 ng/µl) were added. The candidate F1 animals were identified by following a roller phenotype. The F2 progenies were isolated and homozygosed, and PCR and sequencing confirmed the insertion.

### Real-time quantitative PCR (qPCR)

Total RNAs were extracted using the RNeasy Plus Mini Kit (QIAGEN). cDNAs were synthesized using the RevertAid First Strand cDNA Synthesis Kit (Thermo Fisher Scientific). Real-time qPCRs were performed to assay RNAi efficiency using CFX Connect Real-Time PCR Detection System and the iTaq Universal SYBR Green Supermix (Bio-Rad). mRNA levels of target genes were normalized by *act-5* mRNA. qPCR was performed with the following primers: *act-5*, 5′-CAA​CAT​TCA​GGC​TGT​GCT​TT-3′ and 5′-TGA​TGG​ATT​GGT​AGG​TGG​TCT-3′; *age-1*: 5′-ATG​GAG​CAA​GGA​TAG​GCA-3′ and 5′-ACG​ACT​CAT​AGT​CAT​CAC​CC-3′; *ppk-1*: 5′-CAC​AAC​CCA​AAC​TTC​TAC​GC-3′ and 5′-TCC​TGT​TCT​TCG​GGC​ACT-3′; *ppk-2*: 5′-CCA​ATC​GTC​CCG​TAG​AAC​A-3′ and 5′-TGA​TGC​CGT​AGT​AGG​TGA​GA-3′; *ppk-3*: 5′-CGA​AGA​GGG​AAA​GCA​GAC-3′ and 5′-TGA​GCA​TAG​CCG​AGT​TTG-3′; *vps-34*: 5′-TGG​GAA​CAC​GAG​GAT​GAT-3′ and 5′-AAG​ACC​ACG​AGA​TTG​ACG-3′; *pifk-1*: 5′-GGG​GAG​AAT​GAT​ACA​AGC​G-3′ and 5′-GCA​CAA​GAC​GGT​GAA​CTG​A-3′; and *sorb-1*: 5′-TCC​TCC​ACC​ACT​TCC​ACT-3′ and 5′-CGT​CTT​CTC​CCT​TTC​TAT​GC-3′.

### Whole-worm immunoprecipitation

WT and RTKN-1-GFP adult animals were collected (9-cm plates × 10) and washed with M9 buffer. The 100-µl worm pellet was resuspended in 200 µl ice-cold lysis buffer (25 mM Tris-HCl [pH 7.5], 100 mM NaCl, 1 mM EDTA, 1% NP-40 [made from fresh 10% NP-40], 1 mM PMSF) containing protease-inhibitor cocktail (Sigma). The pellet was lysed using an automatic grinding machine (JX-FSTPRP; Jingxin Inc.) at 65 Hz for 15 min (10-s interval after every minute). The lysate was incubated on ice for 30 min and then centrifugated at 13,000 ×*g* for 10 min. The supernatant was incubated with 20 µl GFP-Trap Agarose (Chromotek) overnight at 4°C. Precipitates were washed five times for 10 min with lysis buffer and subjected to immunoblotting using anti-actin and anti-GFP polyclonal antibodies.

### Phalloidin labeling

Animal dissection was performed as previously described (Grant et al., 2001). Dissected samples were fixed in 4% paraformaldehyde (Beyotime) in PBS for 30 min at room temperature, and three times for 10 min with PBS followed by 10 min of treatment with PBS containing 0.5% Triton X-100. Alexa Fluor 488 phalloidin (final 330 nM in PBS; Thermo Fisher Scientific) was used for whole-mount staining for 1 h at room temperature. The specimens were washed three times for 10 min in PBS to remove unbound phalloidin and then subjected to confocal microscopy.

### Confocal microscopy and imaging analysis

A Nikon C2 laser scanning confocal microscope equipped with a 100× (NA 1.4) oil objective was used to acquire the fluorescence signals. Images were captured using NIS-Elements Advanced Research version 4.40.00 software (Nikon). Z-series of optical sections were obtained using 0.6–1.0-µm step size. Metamorph version 7.8.0.0 software (Universal Imaging) was used to examine fluorescence. The “Integrated Morphometry Analysis” function was used to measure the fluorescence area (total area) and the fluorescence intensity (total intensity) within unit regions. A total of 18 cells from nine animals of each genotype were collected for analysis. For colocalization assay, images were captured on young adults; DAPI channel was used to indicate broad-spectrum autofluorescence. From a total of 12 animals of each genotype, Mander’s coefficients and Pearson’s correlation coefficients were acquired using a plugin, JACoP, of the open-source Fiji (ImageJ) software (Schindelin et al., 2012). For tracking the dynamics of endosomal carriers and actin structures, animals were loaded to a Stellaris 5 confocal microscope (Leica) equipped with a 63× (NA 1.4) oil objective and white light lasers. Dynamic images were captured using LAS X software (Leica). Synchronized day 1 adults were selected for imaging (GFP and mCherry channels).

### GST pulldown assay and protein purification

The Gateway BP and LR recombination system were employed to construct expression vectors. The PCR products of rtkn-1a, rtkn-1a(1-231aa), rtkn-1a(232-515aa), rtkn-1a(346-515aa), rtkn-1a(386-515aa), rtkn-1a(232-385aa), rtkn-1a(1-345aa), unc-60a, gsnl-1, cor-1 and pod-1 coding sequences were introduced in frame into entry vector pDONR221 by BP reaction. pGEX-2T destination vector (GE Healthcare Life Sciences) with a Gateway cassette was used for prokaryotic expression. pcDNA3.1 destination vector (Invitrogen) with a 2xHA tag and a Gateway cassette was used for in vitro transcription and translation. LR reactions were conducted to transfer the coding sequences into the above destination vectors. HA-tagged proteins were synthesized in vitro using pcDNA3.1 vectors and a TNT Coupled Transcription/Translation System (Promega). The pGEX-2T vectors were transformed into the ArcticExpress strain of *E. coli* (Stratagene) to express GST fusion proteins. The bacteria were pelleted and lysed in lysis buffer (50 mM Hepes [pH 7.5], 400 mM NaCl, 1 mM DTT, 1 mM PMSF) supplemented with Complete Protease Inhibitor Mixture Tablets (Sigma). Extracts were cleared by centrifugation (10,000 rpm for 60 min), and supernatants were incubated with 300–3,000 µl glutathione sepharose 4B beads (GE Healthcare Life Sciences) at 4°C overnight. Beads were washed five times with cold STET buffer (10 mM Tris-HCl [pH 8.0], 150 mM NaCl, 1 mM EDTA, 1mM DTT, 0.2% [vol/vol] NP-40) and three additional times with 50 mM Tris-HCl (pH 8.0). GST-tagged proteins were eluted by using 50 mM Tris-HCl (pH 8.0) containing 10 mM glutathione. After centrifugation (13,000 rpm for 30 min at 4°C), eluted proteins were further purified with the AKTA system equipped with Superdex 200 or Superose 6 size exclusion chromatography (GE Healthcare Life Sciences). For GST pulldown assays, 20-µl beads were incubated with purified proteins and washed five times with cold STET buffer, then incubated with HA-tagged proteins overnight at 4°C. Beads were washed five times in cold STET buffer to remove unbound proteins; 20 µl 2xSDS-PAGE loading buffer was then added to each sample. After boiling, eluted proteins were resolved on SDS-PAGE (10% [wt/vol] polyacrylamide) and blotted onto nitrocellulose. The blot was probed with anti-HA or anti-GST antibody.

### F-actin cosedimentation assay

F-actin binding potential was assayed using an Actin-Binding Protein Biochem Kit: Non-Muscle Actin (BK013; Cytoskeleton). G-actin was prepared by dilution with general actin buffer to 1 mg/ml. Actin polymerization buffer was added into G-actin preparations. F-actin was obtained by incubation of the reaction cocktail for 1 h at room temperature. The purified GST-tagged proteins were centrifuged at 150,000 x*g* for 1 h at 4°C, and the supernatant was used for F-actin cosedimentation. GST-tagged proteins were incubated with an equal volume of general actin buffer or F-actin for 30 min and then centrifuged at 150,000 x*g* for 1.5 h at room temperature. For actin-bundling detection, F-actin was incubated with purified proteins for 30 min and centrifuged at 15,000 x*g* for 1 h at room temperature. The pellet/supernatant ratio was quantified by densitometry using FluorChem FC3 version 3.4.0 (ProteinSimple).

### F-actin polymerization and depolymerization assays

Actin Polymerization Biochem Kit: Muscle Actin (BK003; Cytoskeleton) was used for both polymerization and depolymerization assays. The fluorescence intensity of the pyrene-conjugated actin was measured to monitor actin polymerization or depolymerization dynamics. In actin polymerization assay, 10 µM pyrene-G-actin was prepared by the standard protocol. The purified GST-tagged proteins were added into pyrene-G-actin preparation. The fluorescence intensity was recorded every minute over 20 min using a Synergy 2 modular multimode reader (BioTek Instruments) at an excitation wavelength of 350 nm and an emission wavelength of 407 nm. The purpose of this step is to detect whether the protein itself has a polymerization activity. Then, actin polymerization buffer was added to the reaction, and the fluorescence intensity was recorded for an additional 100 min. For actin depolymerization assay, 5 µM pyrene-G-actin was used for the preparation of F-actin. F-actin was diluted to 1 µM, and the fluorescence intensity was recorded every minute over 3 min to obtain the baseline. The purified GST-tagged proteins were then added into pyrene-F-actin preparation, and the fluorescence signal was recorded for an additional 60 min.

### Crosslinking protein interaction analysis and native PAGE

The cleavage site of PreScission protease (LEVLFQGP) followed by the HA coding sequence was inserted behind the GST in the pGEX-2T vectors via overlapping PCR. GST fusion proteins with the PreScission protease cleavage site were expressed in the ArcticExpress strain of *E. coli* (Stratagene). Bacterial pellets were lysed, and supernatants were collected and incubated with glutathione sepharose 4B beads as described previously. After five 10-min washes with cold STET buffer, the beads were further washed three times with PreScission cleavage buffer (50 mM Tris-HCl, 150 mM NaCl, 1 mM EDTA, 1 mM DTT [pH 7.5]). A total of 50 µg GST-PreScission Protease was added into 300-µl beads per sample. The cleavage reaction was performed at 4°C overnight. The supernatant was acquired by centrifugation (13,000 rpm for 30 min at 4°C), and the protein concentration was assessed. After centrifugation (13,000 rpm for 30 min at 4°C), eluted proteins were further purified with the AKTA system equipped with Superdex 200 (for HA-RBD-PH) or Superose 6 (for HA-RTKN-1) size exclusion chromatography (GE Healthcare Life Sciences). For crosslinking analysis, proteins were covalently crosslinked for 30 min at 25°C in PBS (disuccinimidyl glutarate, #20593; Thermo Fisher Scientific) and incubated with 20 mM Tris for 15 min to terminate the reaction. Proteins were separated on SDS-PAGE using a 3%, 5%, and 8% (wt/vol) gradient resolving gel and stained with Coomassie blue. For native gel analysis, proteins (20 µg) were mixed with 5× Native Gel Sample Loading Buffer (Beyotime). Samples were separated on native PAGE (3–12% Bis-Tris Protein Gel; Thermo Fisher Scientific) and stained with Coomassie blue.

### Statistical analysis

Statistical analyses in this study were performed and plotted using GraphPad Prism 8.02 software (GraphPad Software). Biochemical datasets were analyzed by *t* test, and Mann–Whitney *U* test was used for most imaging data sets. For comparison of more than two data sets, one-way ANOVA followed by a Dunn’s post hoc multiple comparison test was applied.

### Online supplemental material

Fig. S1 shows the measurement of growth time of WT and *rtkn-1(ok1404)* mutant animals. Also shown are the actin integrity in WT and mutant animals and the colocalization between GFP-Utrophin-CH and endogenous RTKN-1. Fig. S2 shows the colocalization between RTKN-1 or RTKN-1-PH and endosome markers. Fig. S3 shows the localization of CIE recycling cargo hTAC-GFP, CDE nonrecycling cargo GFP-CD4-LL, CDE recycling cargo hTfR-GFP, CDE retrograde cargo MIG-14-GFP, apical recycling cargo LRP-1-GFP, and recycling endosome markers ARF-6-GFP and SDPN-1-GFP. Fig. S4 shows the endogenous level of UNC-60A or RTKN-1 and the colocalization between UNC-60A and PI(4,5)P2. Also shown are the effects of GST-COR-1 or GST-POD-1 on UNC-60A–mediated actin depolymerization, the GST-RTKN-1 pulldown assay with HA-UNC-60A, and distribution of RTKN-1-GFP in *unc-60a(RNAi)* animals. Fig. S5 shows the GST pulldown assay of RTKN-1 truncations and native PAGE of HA-RBD-PH and HA-RTKN-1. Also shown are the actin-bundling assay of HA-RTKN-1; the localization of RTKN-1-GFP, EHBP-1-GFP, and GFP-Utrophin-CH in indicated genetic backgrounds; and the distribution of hTAC-GFP in *sorb-1(RNAi)* animal. Video 1 is a time-lapse analysis of hTAC-mCherry–labeled endosomal dynamics in a WT animal coexpressing GFP-Utrophin-CH. Video 2 is a time-lapse analysis of hTAC-mCherry–labeled endosomal dynamics in an *rtkn-1(ok1404)* mutant animal coexpressing GFP-Utrophin-CH. Table S1 lists the strains and transgenes used and created in this study.

## Supplementary Material

Table S1 lists the strains and transgenes used and created in this study
